# IQSEC2 mutation associated with epilepsy, intellectual disability, and autism results in hyperexcitability of patient-derived neurons and deficient synaptic transmission

**DOI:** 10.1038/s41380-021-01281-0

**Published:** 2021-09-17

**Authors:** Boris Brant, Tchelet Stern, Huda Adwan Shekhidem, Liron Mizrahi, Idan Rosh, Yam Stern, Polina Ofer, Ayat Asleh, George K. Essien Umanah, Reem Jada, Nina S. Levy, Andrew P. Levy, Shani Stern

**Affiliations:** 1 Sagol Department of Neurobiology, Faculty of Natural Sciences, University of Haifa, Haifa, Israel; 2 Department of Neurology, Johns Hopkins University, Baltimore, MD USA; 3 Technion Faculty of Medicine, Technion-Israel Institute of Technology, Haifa, Israel

**Keywords:** Stem cells, Neuroscience

## Abstract

Mutations in the *IQSEC2* gene are associated with drug-resistant, multifocal infantile and childhood epilepsy; autism; and severe intellectual disability (ID). We used induced pluripotent stem cell (iPSC) technology to obtain hippocampal neurons to investigate the neuropathology of *IQSEC2*-mediated disease. The neurons were characterized at three-time points during differentiation to assess developmental progression. We showed that immature *IQSEC2* mutant dentate gyrus (DG) granule neurons were extremely hyperexcitable, exhibiting increased sodium and potassium currents compared to those of CRISPR-Cas9-corrected isogenic controls, and displayed dysregulation of genes involved in differentiation and development. Immature *IQSEC2* mutant cultured neurons exhibited a marked reduction in the number of inhibitory neurons, which contributed further to hyperexcitability. As the mutant neurons aged, they became hypoexcitable, exhibiting reduced sodium and potassium currents and a reduction in the rate of synaptic and network activity, and showed dysregulation of genes involved in synaptic transmission and neuronal differentiation. Mature *IQSEC2* mutant neurons were less viable than wild-type mature neurons and had reduced expression of surface AMPA receptors. Our studies provide mechanistic insights into severe infantile epilepsy and neurodevelopmental delay associated with this mutation and present a human model for studying *IQSEC2* mutations in vitro.

## Introduction

Mutations in *IQSEC2*, an X-linked gene, are associated with intellectual disability (ID), autism, and epilepsy [1–3]. Seventy different mutations in *IQSEC2* that are associated with moderate to severe ID, epilepsy, and autistic traits have been described [3]. Understanding the molecular pathophysiology of specific *IQSEC2* mutations may allow for a personalized treatment program to provide much-needed hope and help affected children and their families.

The *IQSEC2* gene contains an IQ-like calmodulin-binding motif and a SEC7 homology domain responsible for the guanine nucleotide exchange factor (GEF) catalytic activity of *IQSEC2*. *IQSEC2* can promote the exchange of guanosine diphosphate (GDP) for guanosine triphosphate (GTP) on ADP ribosylation factors (ARFs), thereby resulting in activation of the ARF. *IQSEC2* has an apparent specificity for *ARF6*, which is a critical regulator of membrane trafficking and synaptic structure and function in neurons [1, 4, 5]. The IQ motif has been proposed to regulate the GEF catalytic activity of *IQSEC2* in a calcium-dependent fashion. The protein product of *IQSEC2* is localized to excitatory glutamatergic synapses as part of the NMDA receptor complex and has been proposed to regulate AMPA receptor trafficking and synaptic plasticity.

Missense mutations, nonsense mutations, splice site mutations, deletions, indels, duplications, and structural variants in the *IQSEC2* gene have been described [3]. A precise genotype-phenotype correlation has not been identified to date. Children with disease-causing *IQSEC2* mutations exhibit epilepsy, ID, autism, developmental regression, and language deficits. However, the nature and severity of these phenotypes appear to differ between individuals depending on the different pathogenic variants, the way they were inherited, and the gender of the individual with the variant. For instance, although *IQSEC2* was initially demonstrated to be a candidate gene for X-linked neurologic disorders in males, new emerging case reports of heterozygote females with *de novo* loss of function of a single *IQSEC2* allele have reported severe phenotypes, which might indicate more complicated X-linked inheritance patterns. These conclusions were reaffirmed in a recently published study on *IQSEC2* knockout male and heterozygote female mice recapitulating the epileptic, autistic, and ID phenotypes observed in both male and female patients [3]. Differences were observed between males and females in patients with missense functional *IQSEC2* variants, with males demonstrating mild to severe nonsyndromic ID accompanied by seizures and autistic traits and females with missense functional *IQSEC2* mutations being generally either asymptomatic carriers or mildly affected, exhibiting borderline ID. Another *IQSEC2* mutation, a novel heterozygous *IQSEC2* frameshift variant in an asymptomatic mother with two sons who are hemizygous carriers of the variant and exhibit severe delayed psychomotor development and progressive epilepsy, was recently reported [6]. Recently, missense mutations in amino acid residue 350 of *IQSEC2* associated with ID, epilepsy, and autism were described [7–9]. In a mouse model with the A350V *IQSEC2* mutation generated by CRISPR, the molecular pathophysiology associated with the mutation was AMPAR-deficient trafficking, and the mice displayed abnormal behavioral and cognitive phenotypes [10] as well as seizures.

In this study, we used induced pluripotent stem cell (iPSC) technology to characterize the effect of the A350V *IQSEC2* mutation on patient-derived human hippocampal neurons compared to CRISPR-Cas9-corrected isogeneic neurons in which the A350V *IQSEC2* mutation was corrected back to the wild-type *IQSEC2* sequence. This characterization of hippocampal neurons was performed over a long period of neuronal differentiation. We showed that young, A350V *IQSEC2* immature neurons—were extremely hyperexcitable and exhibited decreased inhibition and dysregulation of genes involved in differentiation and development. As *IQSEC2* mutant neurons aged, they developed synaptic deficits compared to CRISPR-Cas9-corrected neurons. RNA sequencing coupled with surface protein cross-linking and immunohistochemical analysis revealed that these synaptic changes were accompanied by dysregulation of synaptic genes and notable downregulation of surface AMPA receptor expression. Our work is a thorough investigation and characterization of the pathophysiological changes in these *IQSEC2* mutant hippocampal neurons and provides a platform for the development of precision medicine-based therapeutics for a disease for which there are currently no treatments.

## Materials and methods

### Generation of iPSCs

After obtaining institutional committee approval and parental written informed consent, we generated iPSCs from the fibroblasts of a male child hemizygous for the A350V *IQSEC2* [9] mutation by transfection with Sendai virus vectors. Immunohistochemistry of the iPSCs confirmed the expression of appropriate iPSC markers. Gene editing of the patient-derived iPSCs to restore the wild-type *IQSEC2* genotype (i.e., converting the valine residue at site 350 back to an alanine residue) was successfully achieved by CRISPR-Cas9 technology, and these cells served as wild-type controls with the same genetic background as the mutant *IQSEC2* cells. Supplementary Fig. S8 shows the method used for the characterization of the iPSC lines and the corrected isogenic control line.

### Neuronal cultures

DG granule neurons were plated according to a previously described protocol [10]. Briefly, iPSCs were grown to ~80% confluence and dissociated into embryoid bodies (EBs) using collagenase IV. EBs were grown in ultralow attachment plates in an anti-caudalizing medium containing DMEM/F12, Glutamax, B27 (-vitamin A), N2, Noggin, *DKK1*, cyclopamine, and SB431542. The medium was changed 3 times a week. On day 20, the EBs were plated on poly-L-ornithine/laminin-coated plates with DMEM/F12, Glutamax, B27 (-vitamin A), N2, and laminin. On day 27, neural rosettes were selected according to morphology and dissociated using Accutase. The dissociated rosettes were plated as neural progenitor cells (NPCs) on poly-l-ornithine/laminin-coated plates in DMEM/F12, Glutamax, B27 (-vitamin A), N2, laminin, and *FGF2*. The NPCs were grown until they reached 40% confluence and then differentiated into neurons in a differentiation medium containing DMEM/F12, Glutamax, N2, B27 (-vitamin A), ascorbic acid, *BDNF*, Wnt3A, laminin, and *cAMP*. To enhance neuronal maturation and connectivity, we dissociated the cultured neurons 14 days after the start of differentiation and cultured them in BrainPhys medium [11] 21 days after the start of differentiation. The neurons were infected with a *PROX1*::*GFP* lentivirus [10] on day 17. CRISPR-Cas9-edited iPSCs were differentiated into neurons in parallel to the unedited iPSCs to identify A350V *IQSEC2* mutation-specific neuronal changes.

### Electrophysiology

The whole-cell patch-clamp recordings were performed at three different time points: 5 weeks, 7 weeks, and 11 weeks after the start of differentiation. The neurons were infected with a *PROX1*∷*eGFP* lentiviral vector at 17 days post-differentiation. Only GFP-positive neurons were patched as previously described [12], as GFP-labeled neurons expressing *PROX1*, were DG granule neurons (see Supplementary Methods).

### Analysis of electrophysiological recordings

Analysis was performed as previously described [12] (see Supplementary Methods).

### Immunohistochemistry

Cells were fixed in 4% paraformaldehyde for 15 min. The cells were subsequently blocked and permeabilized in PBS containing 0.1–0.2% Triton X-100 and 10% horse serum. Coverslips were incubated with the primary antibody in blocking solution overnight at 4 °C, washed in Tris-buffered saline, incubated with secondary antibodies for 30 min at room temperature, counterstained with DAPI, washed, mounted on slides using Fluoromount-G (Southern Biotech), and dried overnight away from light. The following antibodies were used at the indicated dilutions: *MAP2ab* (1:500), *PROX1* (1:500), *GABA* (1:500), and *Caspase3* (1:200). Fluorescence signals were detected using a Zeiss 710 confocal microscope, and images were processed with Zen and ImageJ software.

### FACS sorting

Neurons were chemically dissociated with Accutase and then mechanically dissociated. The dissociated cells were resuspended in DPBS containing Zombie Violet. The neurons were then placed on ice for 10 min protected from light. After 10 min, the solution containing the cells was centrifuged, the supernatant of the solution was aspirated, and the neurons were resuspended in DPBS with knockout serum (KSR) (0.5%) and 1:1000 Rock inhibitor. The neurons were then filtered and transferred to a FACS tube to sort *GFP*-labeled *PROX1* neurons and live neurons, which were not stained with Zombie Violet. The neurons were then sorted in TRIzol LS and kept at −80 °C until RNA preparation.

### RNA preparation

RNA was prepared using the Zymo RNA Clean and Concentrator kit following the manufacturer’s instructions.

### RNA sequencing and analysis of DG granule neurons

RNA was collected from control and *IQSEC2* mutant DG granule neurons at three different time points after differentiation. Libraries for the 5- and 7-week time points were prepared using a TruSeq RNA Library Prep Kit v2. At 11 weeks, samples were prepared using a SMART-Seq v4 Ultra Low Input RNA Kit according to the manufacturer’s instructions (Illumina). Further information is provided in the Supplementary Methods.

### Surface protein crosslinking assay for the detection of surface AMPA receptors in wild-type DG and *IQSEC2* mutant neurons

To determine the relative distribution of surface AMPA receptors in *IQSEC2* mutant neurons compared to wild-type DG granule neurons, a surface protein-crosslinking assay was performed using the membrane-impermeable crosslinking agent bis (sulfosuccinimidyl) suberate (BS_3_, Sigma) as previously described [13] with some modifications. BS_3_ is a membrane-impermeable crosslinking agent that selectively crosslinks cell-surface proteins, forming high-molecular-mass aggregates. Non crosslinked intracellular proteins retain their normal molecular mass. Wild-type and *IQSEC2* mutant *PROX1*-expressing DG granule neurons were treated with ice-cold PBS buffer with or without 2 mM BS_3_ and then incubated for 3 hours at 4 °C with gentle agitation. The reaction was quenched with 0.1 M glycine in PBS (10 min, 4 °C), and the cells were lysed in ice-cold lysis buffer (PBS containing 1% Triton X-100, 0.5% SDS, 5 mM EDTA (pH 7.4), and protease inhibitor cocktail). The lysates were homogenized and centrifuged at 15,000×*g* for 5 min. The total protein concentration in the supernatant was determined. Approximately 10 μg of total protein from each sample was resolved by 10% SDS-PAGE, and western immunoblotting was performed to analyze the surface and intracellular expression of AMPA receptors using anti-*GluA2* (rabbit monoclonal, Abcam Ab150387) (1:2000) and HRP-conjugated monoclonal mouse anti-beta-actin (Millipore-SIGMA, A3854) (1:5,000) antibodies. The signals on the blots were visualized with Femto-HRP Chemiluminescent Substrate Kits (1B1583, AMRESCO) and imaged with an Amersham Imager 600 instrument. The band intensities on all blots were measured using NIH ImageJ software (Rasband, W.S., NIH, http://rsb.info.nih.gov/ij/). All experiments were performed with 3–4 biological replicates, and quantitative data are presented as the mean ± standard error of the mean (SEM) and were analyzed by GraphPad Prism 6 software (Instat, GraphPad Software). Statistical significance was assessed by *t*-test (two-tailed). Differences were considered significant at *p* < 0.05.

### Immunocytochemistry for surface AMPA receptors in wild-type DG and *IQSEC2* mutant granule neurons

DG granule cells were fixed with 4% PFA in PBS, washed three times with PBS, and blocked with 2.5% normal goat serum in PBS for 30 min at 4 °C. To label surface *GluA2*, sections were incubated at 4 °C overnight in PBS containing 0.5% normal goat serum and a mouse monoclonal Alexa Fluor 488-conjugated extracellular anti-N-terminal *GluA2* antibody (Millipore-Sigma, MAB397A4). After four washes with PBS, the cells were permeabilized with 0.3% Triton X-100 and 2.5% normal goat serum in PBS and incubated with a rabbit monoclonal intracellular anti-C-terminal *GluA2* antibody (Abcam, Ab150387) to label total *GluA2* for 4 hours. The cells were then incubated in PBS containing an Alexa Flour 555-conjugated goat anti-rabbit IgG secondary antibody (Thermo Fisher Scientific, A32732) for 1 hour at room temperature and then stained with DAPI for 5 min. After four washes with PBS, the sections were mounted on precleaned slides with Immuno-Mount (Thermo Scientific). Images were acquired using a Zeiss LSM laser-scanning confocal microscope. Images for all conditions in the individual experiments were analyzed using identical acquisition parameters, and identical values were used as thresholds. The fluorescence intensities of the labeled surface and internalized receptors were measured using Zen software (Zeiss). Total and surface expression was normalized to the DAPI signal. The data are presented as the mean ± SEM. The significance of the difference in average fluorescence intensity between groups was analyzed by *t*-test (two-tailed), and *p* < 0.05 was considered statistically significant.

### RNA sequencing of hippocampal tissues from A350V *IQSEC2* and wild-type mice

RNA sequencing of RNA extracted from the hippocampi of A350V *IQSEC2* and wild-type mice was performed on postnatal day 16. Further information is provided in the Supplementary Methods.

## Results

### *IQSEC2* mutant neurons are hyperexcitable at 5–6 weeks

**Fig. 1:**
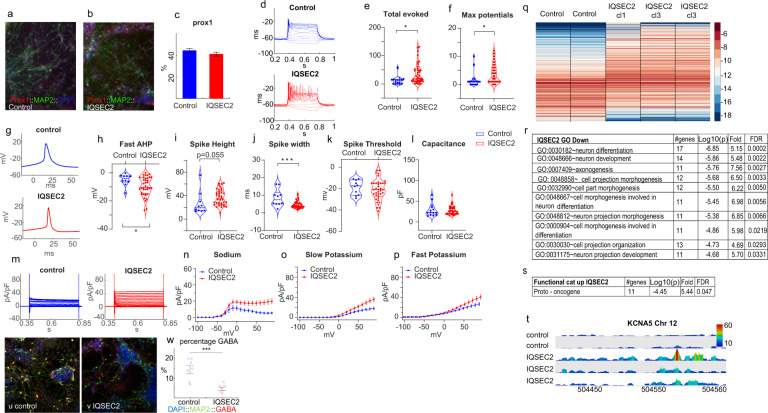
Young (5 weeks post differentiation) *IQSEC2*-mutant neurons are hyperexcitable compared to control neurons with a reduction in the number of GABA expressing neurons. **a**, **b** Representative images of a control (**a**) and *IQSEC2*-mutant (**b**) neuronal cultures that were immunostained for DAPI, *MAP2*, and *PROX1*. **c** There was no significant change between the percentage of *PROX1* positive neurons in the control and *IQSEC2*-mutant cultures. **d** A representative recording of evoked potentials in current-clamp mode. **e** The total number of evoked action potentials is larger in the *IQSEC2*-mutant neurons than in the control neurons. **f** The maximum number of evoked action potentials is larger in *IQSEC2*-mutant neurons than in control neurons. **g** Representative traces of a control (upper) and *IQSEC2*-mutant (lower) action potentials. The first action potential evoked with minimal injected current is plotted. **h** The amplitude of the fast AHP is larger in *IQSEC2*-mutant neurons than in control neurons. **i** The spike amplitude is larger in *IQSEC2*-mutant neurons than in control neurons (*p* = 0.055). **j** The spike width is narrower in *IQSEC2*-mutant neurons than in control neurons. **k** The threshold for eliciting an action potential is not different between *IQSEC2*-mutant and control neurons. **l** The cell capacitance is not different between *IQSEC2*-mutant and control neurons. **m** Representative traces of sodium and potassium currents recorded in voltage-clamp in control (left) and *IQSEC2*-mutant (right) neurons. **n** The average sodium currents in *IQSEC2*-mutant neurons are increased compared to control neurons. **o** The average slow potassium currents in *IQSEC2*-mutant neurons are increased compared to control neurons. **p** The average fast potassium currents are increased in *IQSEC2*-mutant compared to control neurons. **q** A heat-map of the differentially expressed genes (by RNA sequencing) between control (2 biological replicates) and *IQSEC2*-mutant DG granule neurons (one sample derived from clone 1 and 2 biological replicates derived from clone 3). **r** Significant gene ontology (GO) terms (Biological processes) that were downregulated in *IQSEC2*-mutant neurons. **s** Functional categories that were upregulated in *IQSEC2*-mutant neurons. **t** A screenshot of *KCNA5* expression; (from the top track down): control neurons sample 1, control sample 2, *IQSEC2*-mutant neurons (clone 1), *IQSEC2*-mutant neurons (clone 3) sample 1, and *IQSEC2*-mutant neurons (clone 3) sample 2. *KCNA5* mRNA is expressed at a significantly higher level in *IQSEC2*-mutant compared with the control neuronal samples. The data was collected using a Wiggle plot with a step size of 2 base pairs. **u** An example image of immunohistochemistry staining for DAPI (blue), *MAP2* (green), and GABA (red) of a control neuronal culture. **v** An example image of immunohistochemistry staining for DAPI (blue), *MAP2* (green), and GABA (red) of an *IQSEC2*-mutant neuronal culture. **w** The averages of the percentage of GABA expressing neurons (identified by the red staining) out of the total neurons (identified with the green *MAP2* staining) in the *IQSEC2*-mutant neuronal cultures are significantly lower than the percentage of GABA expressing neurons in the control neuronal cultures. Data was obtained by imaging 7 control and 7 *IQSEC2*-mutant neuronal cultures. In this figure asterisks represent statistical significance by the following code: *p value<0.05, **p value<0.01, ***p < 0.001, ****p < 0.0001. Error bars represent the standard error in this figure.

Five to six weeks after the differentiation of *IQSEC2* mutant and CRISPR-Cas9 corrected isogenic control iPSCs into dentate gyrus (DG) granule neurons was initiated, we performed whole-cell patch-clamp experiments as well as immunohistochemistry and RNA sequencing. The cultures were infected with a *PROX1::eGFP* lentivirus to label DG granule neurons. Approximately 45% of the neurons in the control cultures were *PROX1*-positive, and 41% of the neurons in the *IQSEC2* mutant cultures were *PROX1*-positive (Fig. 1a is an image of an immunostained control culture, Fig. 1b is an image of an immunostained *IQSEC2* mutant culture, and Fig. 1c shows the average percentage of *PROX1-*positive neurons in 7 control and 7 *IQSEC2* mutant cultures). We obtained whole-cell patch-clamp recordings from a total of 14 control neurons and 37 I*QSEC2* mutant neurons (derived from two different clones). We observed that *IQSEC2* mutant neurons were markedly more hyperexcitable than control neurons, as indicated by two measures: the total number of evoked potentials and the maximum number of potentials (see “Materials and methods”). The total number of evoked potentials was 35 ± 5 in *IQSEC2* mutant neurons and 12 ± 3 for control neurons (*p* = 0.01) (Fig. 1d shows representative traces and Fig. 1e shows the complete statistical analysis). The maximum number of evoked potentials was 3.8 ± 0.5 for the *IQSEC2* mutant neurons and 1.7 ± 0.7 for control neurons (Fig. 1f, *p* = 0.03). Interestingly, we also observed changes in spike shape. A representative trace of the first evoked spike (see “Materials and methods”) is shown in Fig. 1g. The fast AHP was larger in the *IQSEC2* mutant neurons (Fig. 1h, −4.9 ± 1.3 mV (control) vs. −9.8 ± 1.1 mV (*IQSEC2* mutant), *p* = 0.035). The spike height was almost significantly larger in the *IQSEC2* mutant neurons than in the controls (Fig. 1i, 26.6 ± 6.1 mV) (control) vs. 37 ± 2.2 mV (*IQSEC2* mutant, *p* = 0.055). The spike width was narrower in the *IQSEC2* mutant neurons (Fig. 1j, 8.5 ± 1.5 ms (control) vs. 4.6 ± 0.3 ms (*IQSEC2* mutant), *p* = 0.0003). The spike threshold was not significantly different between the *IQSEC2* mutant neurons and the control neurons (Fig. 1k). The capacitance of the *IQSEC2* mutant neurons was not significantly different from that of the control neurons (Fig. 1l). The sodium currents and the potassium currents (both fast and slow) were larger in the *IQSEC2* mutant neurons (representative traces are presented in Fig. 1m, the average sodium current is shown in Fig. 1n, the slow potassium current is presented in Fig. 1o and the fast potassium current is shown in Fig. 1p).

The neuronal cultures were sorted based on *GFP* and Zombie expression to obtain live DG granule neurons (see Methods), and RNA was prepared from the sorted DG granule neurons and sent for sequencing. Two biological replicates from control neurons, two biological replicates from *IQSEC2* mutant neurons derived from clone 3, and one from *IQSEC2* mutant neurons derived from clone 1 were used for sequencing. The results for the two *IQSEC2* mutant clones were pooled together in the analysis. At 5 weeks, there were a total of 361 differentially expressed genes. A heat map of gene expression is shown in Fig. 1q (see “Materials and methods”). Figure 1r presents the gene ontology (GO) terms that were significantly different between the control and *IQSEC2* mutant DG granule neurons. Among the terms enriched for downregulated genes were differentiation and development, and the expression of proto-oncogenes was upregulated in *IQSEC2* mutant neurons (Fig. 1s). One of the genes that were markedly differentially expressed (upregulated with a fold change of 3.4, FDR = 2.2e−4) was *KCNA5*. This is a voltage-gated potassium channel, and the upregulation of its expression is consistent with the increases in both the potassium currents and the fast AHP. It is interesting to note that an increase in the expression of the *KCNA6* gene (which has a similar function) was previously observed in another autism spectrum disorder (ASD) model [14] and in RNA extracted from the hippocampi of IQSEC2 model mice (data not shown). Figure 1t shows the reads from the RNA sequencing results for chromosome 12: 5043954–5046808, which map to the *KCNA5* gene. The complete list of differentially expressed genes is shown in Supplementary Table S1.

### Decreased number of GABA-expressing neurons in *IQSEC2* mutant cultures

It was previously shown that when differentiation into DG granule neurons is induced, ~15% of the neurons in the culture are inhibitory neurons expressing *GABA* [10]. We used *GABA* antibodies to confirm this finding in our cultures (see “Materials and methods”). Indeed, the control cultures contained 14.6 ± 2% *GABA*-expressing neurons. However, the *IQSEC2* mutant cultures contained only 4.7 ± 0.8% *GABA*-expressing neurons. Figure 1u shows an example image of a control neuronal culture that was immunostained for DAPI (blue), *MAP2* (green), and *GABA* (red). Similarly, Fig. 1v shows an example image of an *IQSEC2* mutant neuronal culture. Figure 1w shows the average value for seven images from three different cultures (*p* = 7e−4).

### *IQSEC2* mutant neurons display larger afterhyperpolarization with increased Na/K currents at 7 weeks

**Fig. 2:**
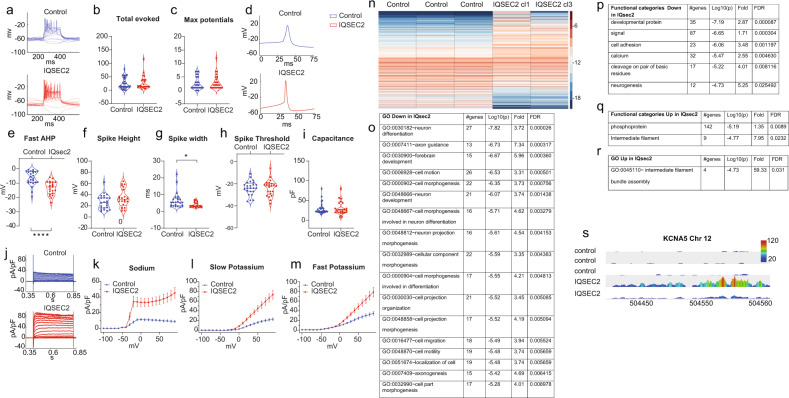
At 7 weeks, *IQSEC2*-mutant neurons start to decrease excitability while control neurons increase excitability compared to the earlier time point. **a** A representative trace of evoked action potentials in the current-clamp mode of control (upper graph) and *IQSEC2*-mutant (lower graph) neurons. **b** The total number of evoked action potentials is similar on average between *IQSEC2*-mutant and control neurons. **c** The maximum number of evoked action potentials is similar between *IQSEC2*-mutant and control neurons. **d** Representative traces of a control (upper graph) and *IQSEC2*-mutant (lower graph) action potentials. The first action potential with minimal injected current is plotted. **e** The amplitude of the fast AHP is larger in *IQSEC2*-mutant neurons than in control neurons. **f** The spike amplitude is similar between *IQSEC2*-mutant neurons and control neurons. **g** The spike width is narrower in *IQSEC2*-mutant neurons than in control neurons. **h** The threshold for eliciting an action potential is not different between *IQSEC2*-mutant and control neurons. **i** The cell capacitance is not different between *IQSEC2*-mutant and control neurons. **j** Representative traces of sodium and potassium currents recorded in voltage-clamp in control (upper graph) and *IQSEC2*-mutant (lower graph) neurons. **k** The average sodium currents in *IQSEC2*-mutant neurons are increased compared to control neurons. **l** The average slow potassium currents in *IQSEC2*-mutant neurons are increased compared to control neurons. **m** The average fast potassium currents are increased in *IQSEC2*-mutant compared to control neurons. **n** A heatmap of the differentially expressed genes (by RNA sequencing) between control (3 biological replicates) and *IQSEC2*-mutant neurons (one sample derived from clone 1 and one sample derived from clone 3). **o** Significant gene ontology (GO) terms (Biological processes) that were downregulated in *IQSEC2*-mutant neurons. **p** Functional categories that were downregulated in *IQSEC2*-mutant neurons. **q** Functional categories that were upregulated in *IQSEC2*-mutant neurons. **r** Significant gene ontology (GO) terms (Biological processes) that were upregulated in *IQSEC2*-mutant neurons. **s** A screenshot of *KCNA5* expression; (from the top track down): control sample 1, control sample 2, control sample 3, *IQSEC2*-mutant clone 1, *IQSEC2*-mutant clone 3 (**s**). *KCNA5* expression is significantly greater in *IQSEC2*-mutant neurons compared to the control neurons. The data was collected using a Wiggle plot with a step size of 2 bp. In this figure asterisks represent statistical significance by the following code: *p value < 0.05, ****p < 0.0001. Error bars represent the standard error in this figure.

Similar to what we have described above, between 7 and 8 weeks after the start of differentiation, we performed patch-clamp experiments and prepared RNA from DG granule neurons. We patch-clamped a total of 36 control neurons and 26 *IQSEC2* mutant neurons (both clones) at this time point (Fig. 2a shows representative recordings). There was no difference in the average excitability of the neurons at this time point. The total number of evoked potentials in control neurons was 24.6 ± 3.6, and that in *IQSEC2* mutant was 26.7 ± 6 (Fig. 2b, *p* = 0.7). The maximum number of evoked potentials was 2.7 ± 0.4 in control neurons and 3.0 ± 0.5 in *IQSEC2* mutant neurons (Fig. 2c, *p* = 0.6). When analyzing the spike shape, we observed a drastic increase in the fast AHP in *IQSEC2* mutant neurons. Representative traces of control and *IQSEC2* mutant spikes are shown in Fig. 2d. The fast AHP data are shown in Fig. 2e (−6.5 ± 1 mV (control) vs. −13.43 ± 1 mV (*IQSEC2* mutant), *p* < 0.0001). The spike amplitude was not significantly different (Fig. 2f, 26.9 ± 2.6 mV (control) vs. 34 ± 3.5 mV (*IQSEC2* mutant), *p* = 0.1). The spike width was narrower in the *IQSEC2* mutant neurons (Fig. 2g, 6.6 ± 1 ms (control) vs. 3.9 ± 0.3 ms (*IQSEC2* mutant), *p* = 0.012). The spike threshold was not significantly different (Fig. 2h, −23.8 ± 1.3 mV (control) vs. −23.8 ± 1.6 mV (*IQSEC2* mutant), *p* = 0.98). The capacitance was not significantly different (Fig. 2i, 28.3 ± 2.2 pF (control) vs. 29.2 ± 3.2 pF (*IQSEC2* mutant), *p* = 0.8). The resting membrane potential was not significantly different between the control and *IQSEC2* mutant DG granule neurons (Supplementary Fig. 13d). Figure 2j shows representative traces of currents recorded from control and *IQSEC2* mutant neurons in voltage-clamp mode. The average currents are plotted in Fig. 2k (sodium), l (slow potassium), and m (fast potassium). The sodium and potassium currents were drastically increased in the *IQSEC2* mutant neurons compared to control neurons.

DG granule neurons were sorted based on *GFP* and Zombie expression 7 weeks after the start of differentiation, and RNA was prepared from the sorted neurons and sent for sequencing. Three biological replicates from control neurons, one sample from *IQSEC2* mutant neurons derived from clone 3, and one sample from *IQSEC2* mutant neurons derived from clone 1 were used for sequencing. The three control samples were pooled together in the analysis, and the two *IQSEC2* mutant samples were pooled together. Figure 2n presents a heat map of the differentially expressed genes (see Methods). There were a total of 691 differentially expressed genes between the *IQSEC2* mutant neurons and the control neurons. Figure 2o presents the significantly dysregulated pathways identified using GO enrichment analysis (molecular pathway). At this time point, neuronal development and differentiation were still among the top pathways enriched for downregulated genes. The functional categories enriched for genes that were downregulated in *IQSEC2* mutant neurons are presented in Fig. 2p, and the functional categories enriched for upregulated genes are presented in Fig. 2q. Only one GO term enriched for upregulated genes was identified: intermediate filament bundle assembly (Fig. 2r). *KCNA5* reads are plotted in Fig. 2s. Consistent with the increase in potassium currents, the expression of the *KCNA5* gene was significantly increased, showing a very large fold change of 12.1 (FDR = 4.2e−5). The complete list of differentially expressed genes at the 7- to 8-week time point is shown in Supplementary Table 2.

### *IQSEC2* mutant neurons are hypoexcitable and have reduced Na/K currents at 11 weeks

**Fig. 3:**
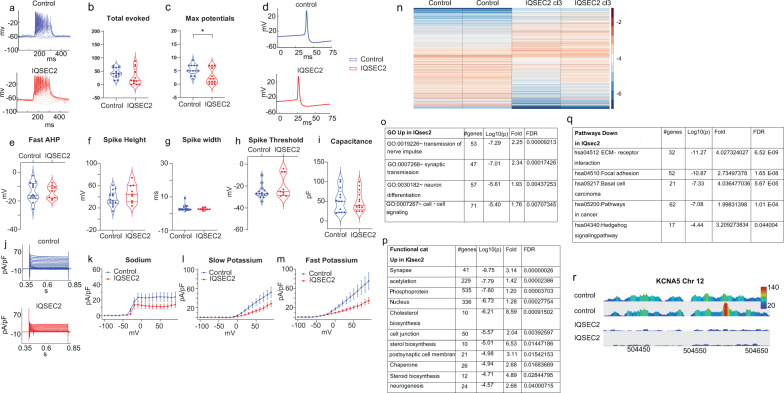
*IQSEC2*-mutant neurons become hypoexcitable at 11 weeks, with reduced sodium and potassium currents. **a** A representative recording of evoked action potentials in the current-clamp mode of control (upper graph) and *IQSEC2*-mutant neurons (lower graph). **b** The total number of evoked action potentials is similar on average between *IQSEC2*-mutant and control neurons. **c** The maximum number of evoked action potentials is reduced in *IQSEC2*-mutant neurons compared to control neurons. **d** Representative traces of action potentials in a control (upper graph) and *IQSEC2*-mutant (lower graph) neurons. The first action potential with minimal injected current is plotted. **e** The amplitude of the fast AHP is similar between *IQSEC2*-mutant and control neurons. **f** The spike amplitude is similar between *IQSEC2*-mutant neurons and control neurons. **g** The spike width is similar between *IQSEC2*-mutant and control neurons. **h** The threshold for eliciting an action potential is not different between *IQSEC2*-mutant and control neurons. **i** The cell capacitance is not different between *IQSEC2*-mutant and control neurons. **j** Representative traces of sodium and potassium currents recorded in voltage-clamp in control (upper graph) and *IQSEC2*-mutant neurons (lower graph). **k** The average sodium currents in *IQSEC2*-mutant neurons are decreased compared to control neurons. **l** The average slow potassium currents in *IQSEC2*-mutant neurons are decreased compared to control neurons. **m** The average fast potassium currents are decreased in *IQSEC2*-mutant compared to control neurons. **n** A heatmap of the differentially expressed genes (by RNA sequencing) between control (2 biological replicates) and *IQSEC2*-mutant neurons (2 biological replicates derived from clone 3). The down-regulated gene ontology terms and functional categories are shown in separate tables (Supplementary Table 3 and Supplementary Table 4). **o** Significant upregulated GO terms in *IQSEC2*-mutant neurons. **p** Functional categories that were upregulated in *IQSEC2*-mutant neurons. **q** Down-regulated KEGGs pathways in *IQSEC2*-mutant neurons compared to control neurons. **r** A screenshot of *KCNA5* expression; (from the top track down): control sample 1, control sample 2, *IQSEC2*-mutant neurons derived from clone 3 sample 1, and *IQSEC2*-mutant neurons derived from clone 3 sample 2. *KCNA5* gene expression at this time point is severely reduced in the *IQSEC2*-mutant samples. The data was collected using a Wiggle plot with a step size of 2 bp. In this figure, asterisks represent statistical significance by the following code: *p value < 0.05. Error bars represent the standard error.

Between 11 and 12 weeks post differentiation, we performed patch-clamp experiments and prepared RNA from DG granule neurons after FACS sorting for *GFP*. We patch-clamped a total of 13 control neurons and 13 *IQSEC2* mutant neurons (clone 3 only, the survival of the *IQSEC2* mutant neurons derived from clone 1 was too low to use them for experiments). The *IQSEC2* mutant neurons were less excitable than the control neurons at this stage. A representative trace of the evoked potentials in the current-clamp mode is presented in Fig. 3a. The results of statistical analysis of the total number of evoked potentials are presented in Fig. 3b (44 ± 5 action potentials (control) vs. 28 ± 9 action potentials (*IQSEC2* mutant), *p* = 0.12). The maximum number of recorded potentials was 5.4 ± 0.5 in control neurons and 2.8 ± 0.8 in *IQSEC2* mutant neurons (Fig. 3c, *p* = 0.014). The spike shape was not significantly different according to all parameters analyzed. The fast AHP was −13.8 ± 1.8 mV in control neurons and −13.7 ± 1.5 mV in *IQSEC2* mutant neurons (Fig. 3e, *p* = 0.97). The spike amplitude was 37.1 ± 3.6 mV in control neurons and 46.4 ± 5.5 mV in *IQSEC2* mutant neurons (Fig. 3f, *p* = 0.15). The spike width was 3.4 ± 0.5 ms in control neurons and 2.7 ± 0.2 ms in *IQSEC2* mutant neurons (Fig. 3g, *p* = 0.3). The threshold for evoking an action potential was −25.4 ± 0.5 mV for control neurons and −19.8 ± 10.5 mV for *IQSEC2* mutant neurons (Fig. 3h, *p* = 0.1). The cell capacitance was 52.7 ± 8.3 pF for control neurons and 51 ± 7.1 pF for *IQSEC2* mutant neurons (Fig. 3i, *p* = 0.88). A representative trace of the currents recorded in voltage-clamp mode is shown in Fig. 3j. The average sodium currents are presented in Fig. 3k, the average slow potassium currents are presented in Fig. 3l, and the average fast potassium currents are presented in Fig. 3m. Overall, there was a reduction in the amplitude of the sodium and potassium currents in the *IQSEC2* mutant neurons compared to the control neurons at this time point, which is the opposite change as that observed at the previous time points.

DG granule neurons were sorted based on *GFP* and Zombie expression (see “Materials and methods”) 11 weeks after the start of differentiation, and RNA was prepared from the sorted neurons and sent for sequencing. Two biological replicates from control neurons and two samples from *IQSEC2* mutant neurons derived from clone 3 were sent for sequencing. The two control samples were pooled together in the analysis, and the two *IQSEC2* mutant samples were pooled together. At this time point, the expression pattern between the *IQSEC2* mutant and control neurons was more marked, with 3055 differentially expressed genes. A heatmap of the differentially expressed genes is plotted in Fig. 3n. The GO pathways enriched for genes that were upregulated are presented in Fig. 3o. The top terms were the transmission of nerve impulses and synaptic transmission. The functional categories enriched for dysregulated genes are presented in Fig. 3p, with the top pathway being synapse. The KEGG pathways enriched for downregulated genes are presented in Fig. 3q and included focal adhesion and ECM receptor interaction. The GO terms enriched for downregulated genes are presented in Supplementary Table 3 and included several pathways that are related to the extracellular matrix and cell adhesion, and the functional categories enriched for downregulated genes are presented in Supplementary Table 4. Figure 3r presents the mapped reads of the *KCNA5* gene, which was differentially expressed, showing a fold change of 0.176 (FDR = 3.2e−7). It is interesting to note that as the potassium currents decreased (instead of increasing, as at earlier time points), the expression level of *KCNA5* also changed, decreasing in the *IQSEC2* mutant neurons compared to the control neurons. The complete list of differentially expressed genes at the 11–12 week time point can be found in Supplementary Table S5.

### *IQSEC2* mutant neurons display severe synaptic deficits and reduced surface GluA2 expression at 11 weeks

**Fig. 4:**
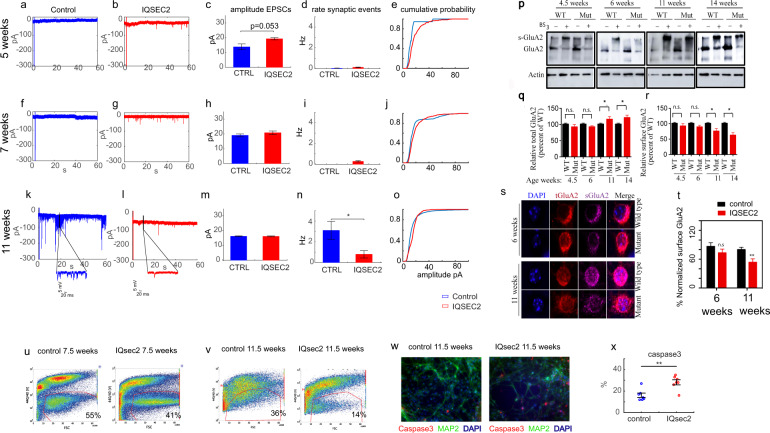
Synaptic transmission is severely impaired and surface *GluA2* is reduced in 11 weeks old *IQSEC2*-mutant neurons. **a** A representative trace of excitatory postsynaptic currents (EPSCs) that were measured in a control neuron at 5 weeks post-differentiation. **b** A representative trace of EPSCs measured in an *IQSEC2*-mutant neuron at 5 weeks post-differentiation. **c** The average amplitude of synaptic events was increased in the *IQSEC2*-mutant neurons (*p* = 0.053). **d** The average rate of synaptic events was not significantly different between control and *IQSEC2*-mutant neurons. **e** The cumulative distribution of the amplitude of synaptic events is right-shifted in the *IQSEC2*-mutant neurons indicating higher amplitudes of synaptic events. **f** A representative trace of synaptic activity measured in a control neuron at 7 weeks of age. **g** A representative trace of synaptic activity measured in an *IQSEC2*-mutant neuron at 7 weeks. **h** The average amplitude of synaptic events is similar between *IQSEC2*-mutant and control neurons at 7 weeks of age. **i** The average rate of synaptic events was not significantly different between control and *IQSEC2*-mutant neurons at 7 weeks. **j** The cumulative distribution of the amplitude of synaptic events shows a slight right shift in *IQSEC2*-mutant neurons. **k** A representative trace of synaptic activity measured in a control neuron at 11 weeks. **l** A representative trace of synaptic activity measured in an *IQSEC2*-mutant neuron at 11 weeks. **m** The average amplitude of synaptic events is similar between *IQSEC2*-mutant and control neurons at 11 weeks post-differentiation. **n** The average rate of synaptic events is significantly decreased in *IQSEC2*-mutant neurons compared to control neurons at 11 weeks. **o** The cumulative distribution of the amplitude of synaptic events. **p** Immunoblots after BS_3_-crosslinking of samples to assess surface (crosslinking “+”) and total protein expression of AMPARs (crosslinking “−”). **q** Quantification of relative total protein levels of AMPA receptor subunits in samples not cross-linked. **r** Quantification of relative surface protein levels of AMPA receptor subunits in samples treated with BS_3_ (crosslinking “+”). Surface expression is the ratio of s-*GluA2* to the total *GluA2* signal in the lane. Signal intensities normalized to actin signal and wild type as 100%. **s** Representative images of labeled total (t*GluA2*) and surface (s*GluA2*) *GluA2* in *IQSEC2*-mutant and control DG granule neurons. **t** Quantification of the percentage of s*GluA2* to t*GluA2* as a measure surface *GluA2* expression. u (left). An image of the FACS gating of a control neuronal culture at 7 weeks after the start of differentiation. The live neurons that passed all the gating (GFP positive indicating PROX1 expression and live) were ~55% of the cells in the culture (marked with the red line). **u** (right). An image of the FACS gating of an *IQSEC2*-mutant neuronal culture at 7 weeks. The live neurons that passed all the gating were ~41% of the cells in the culture. **v** (left). An image of the FACS gating of a control neuronal culture at 11 weeks. The live neurons that passed all the gating were approximately 36% of the cells in the culture. **v** (right) An image of the FACS gating of an *IQSEC2*-mutant neuronal culture at 11 weeks. The live neurons that passed all the gating were approximately 14% of the cells in the culture. **w** (left) Immunohistochemistry staining for DAPI (blue), *MAP2* (green), and Caspase3 (red) in an example control neuronal culture. **w** (right) Immunohistochemistry staining for DAPI (blue), *MAP2* (green), and Caspase3 (red) in an example *IQSEC2*-mutant neuronal culture. **x** An increased average percentage of neurons stained for caspase3 (out of the total *MAP2* positive neurons) in the *IQSEC2*-mutant neuronal cultures compared to the control neuronal cultures indicate an increased number of apoptotic neurons. In this figure, asterisks indicate statistical significance by the following code: *p value < 0.05, **p < 0.01. Error bars represent the standard error.

Using the patch-clamp technique, we did not see differences in the amplitude or rate of synaptic events between *IQSEC2* mutant and control neurons 5–6 weeks after the start of differentiation. Recordings were obtained from a total of 11 control neurons and 39 *IQSEC2* mutant neurons (both from clone 3 and clone 1) at this early time point. Figure 4a shows a representative trace of the synaptic activity (see Methods) of a control neuron, while Fig. 4b similarly shows a representative trace of the synaptic activity of an *IQSEC2* mutant neuron. The overall rate of synaptic activity was low at this stage when the neurons were still immature. The average amplitude of synaptic events was almost significantly larger in the *IQSEC2* mutant neurons (Fig. 4c). The average rate of synaptic events was not significantly different between the *IQSEC2* mutant neurons and control neurons (Fig. 4d). The cumulative distribution of the amplitude of synaptic events is presented in Fig. 4e, which shows a right shift, indicating that the amplitude of synaptic events was larger in the *IQSEC2* mutant neurons.

Similarly, at 7–8 weeks, we did not observe differences in the amplitude or rate of synaptic events between *IQSEC2* mutant and control neurons. Recordings were obtained from a total of 35 control neurons and 25 *IQSEC2* mutant neurons (both from clone 3 and clone 1). At 7–8 weeks, the synaptic rate was still low, indicating that the neurons were still immature. Figure 4f presents a representative trace of the synaptic activity of a control neuron, and Fig. 4g similarly presents a representative trace of synaptic events in an *IQSEC2* mutant neuron. The average amplitude of synaptic events was not significantly different between the control and *IQSEC2*-mutant neurons (Fig. 4h). The average rate of synaptic events was not significantly different between the *IQSEC2* mutant neurons and control neurons (Fig. 4i). The cumulative distribution of the amplitude of synaptic events is presented in Fig. 4j.

However, at 11 weeks post-differentiation, there were dramatic differences in the rate of synaptic events between *IQSEC2* mutant and control neurons, indicating differences in network connectivity. Recordings were obtained from a total of 14 control neurons and 13 *IQSEC2* mutant neurons (from clone 3 only) at this time point. Figure 4k presents a representative trace of the synaptic activity of a control neuron, and Fig. 4l similarly presents a representative trace of the synaptic activity of an *IQSEC2* mutant neuron. While there was no significant difference in the amplitude of synaptic events at this stage (Fig. 4m), there was a drastic and significant reduction in the rate of synaptic events in the *IQSEC2* mutant neuronal network (Fig. 4n). According to the amplitudes of synaptic events, there was no difference in the cumulative distribution of synaptic events between control and *IQSEC2* mutant neurons (Fig. 4o). Additionally, the network activity of the *IQSEC2* mutant neurons was markedly reduced. The duration of the network bursts was reduced by 60% (Supplementary Fig. S13a). The rate of network bursts was decreased by 86% (Supplementary Fig. S13b), and the interburst interval was increased by 600% (Supplementary Fig. S13c).

It was previously shown that mouse hippocampal neurons expressing mutant *IQSEC2* exhibit altered surface *GluA2* AMPA receptor expression [15]. To delineate the effect of the *IQSEC2* mutation in regulating AMPAR trafficking in DG cells, we assessed the level of surface expression of *GluA2* AMPAR in *IQSEC2* mutant and human-derived control DG granule neurons. Cells were fixed at 4.5 weeks, 6 weeks, 11 weeks, and 14 weeks post-differentiation and treated with BS_3_ to assess the surface expression of *GluA2* (Fig. 4p–r). There were no significant differences in the total levels or surface expression of *GluA2* between control and *IQSEC2* mutant cells at 4.5 or 6 weeks. While total *GluA2* expression was significantly higher in mature *IQSEC2* mutant neurons than in control neurons at 11–14 weeks, we observed a decrease in the surface expression of GluA2 in mature *IQSEC2* mutant cells compared to controls cells (similar to what was observed in A350V *IQSEC2* mice [15]). Interestingly, according to RNA sequencing at 11 weeks, the expression of the *GluA2* gene was increased 4-fold (FDR = 0.0015). Immunostaining of mature DG cells further confirmed that surface *GluA2* expression was decreased in *IQSEC2* mutant cells compared to control cells at 11 weeks but not at 6 weeks (Fig. 4s, t).

### Similarities in pathways enriched for differentially expressed genes in patient-derived A350V *IQSEC2* hippocampal neurons and the A350V *IQSEC2* transgenic mouse hippocampus

To further validate the differentially regulated pathways that we identified in human *IQSEC2* mutant hippocampal neurons compared to CRISPR-Cas9-corrected controls by RNA sequencing analysis, we performed RNA sequencing analysis of the A350V *IQSEC2* mouse hippocampus. GO and functional pathway analysis revealed several overlapping dysregulated pathways between the human neurons at 11 weeks post-differentiation (the most mature time point measured in the human neurons) and mouse hippocampal tissue. Interestingly, 60% of the top 5 pathways enriched for downregulated genes identified by GO functional analysis were shared between our mouse and human models, and 40% of the top 5 pathways enriched for upregulated genes identified by GO functional analysis were shared between our mouse and human models (Supplementary Fig. S14). Furthermore, the synapse pathway was the pathway enriched for the most upregulated genes in the human model (3.14-fold enrichment, FDR = 2.6e−7), and the pathway enriched for the second most upregulated genes in the mouse model (7.06-fold enrichment, FDR = 1.4e−11). There were also other overlapping strongly dysregulated synapse-related pathways such as “postsynaptic cell membrane” and “synaptic transmission”. The “calcium” pathway was enriched for downregulated genes in both the human and mouse models (4.96-fold enrichment, FDR = 0.016) (humans); 3.76-fold enrichment, FDR = 7.9e−7 (mice). This analysis provides further validation of our results showing that synaptic and network activity is impaired in the human neuronal model, further suggesting that impaired calcium signaling may play an important role in this pathophysiology, as was previously suggested [16]. The complete list of dysregulated pathways in the hippocampi of *IQSEC2* mutant mice is presented in Supplementary Tables S9–S12.

### *IQSEC2* mutant neurons display increased mortality as they age

When performing FACS sorting for RNA purification, we noticed that there was increased mortality in the *IQSEC2* mutant neurons, which became more marked as the neurons aged. Figure 4u, v presents the FACS plots after gating live neurons (marked with a red line), which were not stained after incubation with Zombie (violet). Figure 4u (left) is a FACS image of control neurons at 7.5 weeks, at which point the percentage of live neurons in the control cultures was 55%. At the same time point, the percentage of live cells in the *IQSEC2* mutant neuronal cultures was only 41% (Fig. 4u, right). At 11.5 weeks, the control neuron survival rate was 36% (Fig. 4v, left), while the *IQSEC2* mutant neuronal survival rate was 14% (Fig. 4v, right). It should be noted that neurons derived from clone 1 did not survive to this age; thus, while the survival rate of neurons derived from clone 3 was 14%; the average survival rate of neurons derived from both clones was even lower. To further test whether there was an increase in apoptosis in these neuronal cultures, we stained for caspase3 (see “Materials and methods”) at 11.5 weeks post-differentiation. A representative image of caspase3 staining in the control cultures is presented in Fig. 4w (left), and a representative image of caspase3 staining in the *IQSEC2* mutant cultures is presented in Fig. 4w (right). The results of statistical analysis of the percentage of caspase3-positive neurons are shown in Fig. 4x. There was a significant increase in the percentage of caspase3-positive neurons in the *IQSEC2* mutant cultures compared to the control cultures. The mRNA expression data also demonstrated a significant fold change of 2.44 (FDR = 0.005) in caspase3 expression at this time point.

### Maturation-dependent changes in *IQSEC2* mutant neurons compared to control neurons

**Fig. 5:**
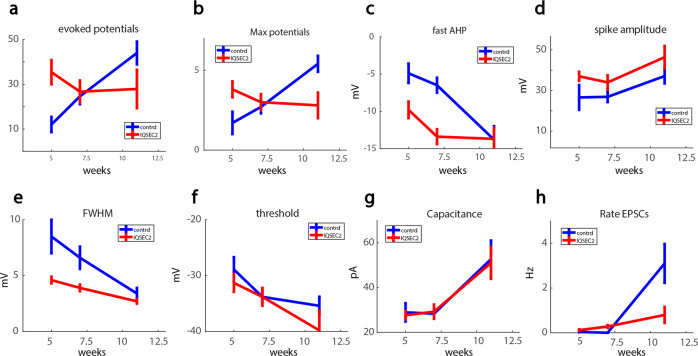
Summary of the progression of changes during the maturation of *IQSEC2*-mutant vs. control neurons. **a** The total number of evoked action potentials is higher in the *IQSEC2*-mutant neurons than the controls at 5 weeks and is decreased at 11 weeks compared to control neurons. **b** Similarly, the Max number of action potentials is higher in the *IQSEC2*-mutant neurons at 5 weeks and later decreases below the controls at 11 weeks. Both these measures in (**a**) and (**b**) represent neuronal excitability. **c** At 5 and 7 weeks the fast AHP amplitude is increased in the *IQSEC2*-mutant neurons, and at 11 weeks the fast AHP is similar between control and *IQSEC2*-mutant neurons. **d** The spike amplitude is larger at all time points in the *IQSEC2*-mutant neurons. **e** The spike width (Full Width at Half the Maximum—FWHM) is narrower in the *IQSEC2*-mutant neurons at 5 weeks and 7 weeks but is similar to the controls at 11 weeks. **f** The spike threshold is similar between the *IQSEC2*-mutant neurons and the control neurons at all-time points. **g** The capacitance is similar between the *IQSEC2*-mutant neurons and the control neurons at all-time points. **h** The rate of excitatory postsynaptic currents (EPSCs) starts very low in both *IQSEC2*-mutant and wild-type neurons and increases significantly at 11 weeks. This rate is reduced in the *IQSEC2*-mutant neurons at 11 weeks when compared to the control neurons.

Figure 5 provides a summary of the electrophysiological features of the *IQSEC2* mutant neurons over the period during which the cells differentiated into hippocampal neurons in culture and developed functional connectivity (5–12 weeks). Initially, *IQSEC2* mutant neurons were hyperexcitable (Fig. 5a, b) compared to the control neurons, but after maturation and by 11 weeks, they not only lost this hyperexcitability but also become hypoexcitable, producing fewer evoked action potentials. Early hyperexcitability was accompanied by an increase in fast AHP (Fig. 5c) and a decrease in spike width (Fig. 5e), both of which were lost when the *IQSEC2* mutant neurons become less excitable at 11 weeks post-differentiation. No significant changes in threshold for eliciting an action potential or capacitance were observed at any of the time points (Fig. 5f–g). Furthermore, when both *IQSEC2* mutant and control neurons were young and immature, they exhibited very little excitatory synaptic activity, but as the neurons matured and developed a network by week 11, the rate of synaptic events was significantly reduced in the *IQSEC2* mutant neurons compared to control neurons (Fig. 5h).

## Discussion

In this study, we used iPSC technology to study patient-derived human neurons with the A350V *IQSEC2* mutation. For our investigation, we chose DG granule neurons since, within the brain, the expression of *IQSEC2* is the highest in the hippocampus. Furthermore, within the hippocampus, the expression is markedly elevated in the DG [5, 16]. Prior work assessing the function of *IQSEC2* has been performed in mouse hippocampal cultures [17] and hippocampal slices [18]. The expression of *IQSEC2* is restricted to excitatory synapses, where it is found in a complex with NMDA receptors in association with PSD-95 in the postsynaptic density [4, 5]. We demonstrated marked differences in the excitability, synaptic connectivity, and survival of *IQSEC2* mutant DG granule neurons compared to CRISPR-Cas9-corrected isogenic control neurons. The early hyperexcitability and reduction in GABA neuronal number may explain infantile epileptic seizures in human subjects, and the reduced synaptic activity in mature neurons may explain ID in patients.

We demonstrated that the increase in the excitability of immature DG granule neurons is associated with increased sodium and potassium currents and a larger afterdepolarization, allowing quick recovery from sodium inactivation. The increase in currents early during differentiation indicates rapid development of *IQSEC2* mutant neurons and is accompanied by dysregulation of neuronal development- and differentiation-related genes. Accelerated development of iPSC-derived neurons from children with autism was shown previously [19]. The large fast AHP is reminiscent of that caused by a mutation in *KCNT1* associated with epilepsy [20], which causes neurons to be hyperexcitable due to increased potassium currents and an increased AHP amplitude. Interestingly, less marked hyperexcitability related to an increased fast AHP amplitude was also found in neurons derived from bipolar disorder patients [12, 21–23], and hypoexcitability was found in hippocampal neurons derived from Down syndrome mice with a reduction in fast AHP [24]. Overall, these studies show that fast AHP has a strong impact on neuronal excitability and is implicated in neurological disorders. The increase in the expression of *KCNA5* in the *IQSEC2* mutant neurons at the earlier time points was coincident with the increases in the potassium current and AHP amplitude and may indicate that this channel is a potential target for mediating an increase in hyperexcitability. Interestingly, the expression of a similar gene, *KCNA6*, was shown to be increased in previous ASD-related studies [14]. An additional factor that we observed in immature *IQSEC2* mutant neuronal cultures that may explain the association of this mutation with epilepsy is the alteration in the excitatory/inhibitory balance caused by a significant reduction in the number of GABA-expressing neurons, and this imbalance may be an additional target for precision medicine.

As *IQSEC2* mutant neurons aged, their excitability decreased compared to that of control neurons. At 11 weeks, they were even less excitable than control neurons. Additionally, their synaptic activity was severely reduced compared to that of control neurons. Interestingly, the most dysregulated pathways were “synaptic transmission” and “transmission of nerve impulse” in our human model, and “synapse” was the second most dysregulated pathway in a validation study on hippocampal tissues from A350V *IQSEC2* model mice. We studied the dysregulated genes involved in these pathways (see Supplementary Table S6 and Supplementary Table S7) and found both presynaptic and postsynaptic genes, indicating that dysregulation occurred at both presynaptic and postsynaptic regions. We hypothesize that the upregulation of these genes is an aspect of a homeostatic mechanism by which the cell tries to compensate for presynaptic mechanisms that reduce the rate of synaptic transmission by increasing the expression of postsynaptic-related genes. It was previously shown that an incorrect dosage of the *IQSEC2* gene results in disrupted dendritic spine morphology [17]. Another possibility is that the mechanisms by which AMPA receptors are brought to the surface are dysfunctional and that the cell tries to compensate for the impairment of these mechanisms by elevating the expression of postsynaptic genes such as *GRIA2* and *GRIA4*. The expression of both of these genes was upregulated at 11 weeks post-differentiation. *GRIA2* was overexpressed, showing a fold change of 4 (*p* = 8.6e−4), and *GRIA4* was overexpressed, showing a fold change of 6 (*p* = 1.2e−11). Overall, we observed dysregulation of the expression of dozens of synaptic genes. This overexpression was evident only at 11 weeks and not at earlier time points. This finding is supported by our immunostaining analysis of the GluA2 *(GRIA2)* protein. When the neurons were young, there were no differences in the expression of either total GluA2 or surface GluA2. However, at 11 and 14 weeks post differentiation, there was an increase in total GluA2 protein expression but a decrease in surface GluA2 expression in *IQSEC2* mutant neurons. We propose that the mechanism by which AMPA receptors are transported to the surface is impaired, causing a marked reduction in synaptic activity. *IQSEC2* mutant cells try to compensate for this reduction in synaptic activity by increasing the production of AMPA receptors. These findings are consistent with previous studies in A350V *IQSEC2* mice [15]. As changes in AMPA receptor density have been indicated to be the structural correlate of learning and memory consolidation, these findings may provide a link between the abnormal cognitive and social-behavioral phenotypes in children with this mutation. Furthermore, these data suggest that altering AMPA receptor trafficking and/or transmission with drugs that increase surface AMPAR expression or drugs that increase surface AMPAR transmission without changing AMPAR receptor density may be a treatment strategy for the ID and social-behavioral defects. AMPA receptor-positive allosteric modulators have been shown to rescue behavioral deficits in autism animal models [25] and are currently in clinical trials for cognitive dysfunction. At this time point, we also observed increased mortality of *IQSEC2* mutant neurons. Previous studies have shown that a reduction in synaptic activity is a cause of neuronal cell death [26–28], which may explain the higher mortality of aged *IQSEC2* mutant neurons.

Numerous mutations in *IQSEC2*, i.e., nonsense mutations, which cause the protein to not be produced (resulting from a premature stop codon), and missense point mutations which occur in either the IQ region (the site where calmodulin binds to *IQSEC2*) or the Sec7 region (the catalytic region that promotes GTP exchange) of *IQSEC2* have been described. The A350V missense mutation described here is found in the IQ region of *IQSEC2*, and several other human missense mutations have been described in this region. As the *IQSEC2* gene is present on the X chromosome, it is expected that the phenotype of a given mutation is affected by gender. Males, which have a single X chromosome, are generally more severely affected by a given mutation (are hemizygous for the mutation) than females who are heterozygotes for X-linked genes. Essentially, all *IQSEC2* mutations are associated with ID, and epilepsy, particularly in males. The precise genotype/phenotype correlation has not yet been fully characterized.

iPSC-derived neurons have been used to study autism, ID, and epilepsy in other genetic disorders. Our findings and previous studies suggest that there may be a common pathophysiological thread in some diseases with different genetic etiologies that may be amenable to a common treatment. iPSC-derived neurons from children expressing multiple different autism- and ID-related genes exhibit hypoexcitability along with decreases in the numbers of inducible and spontaneous ESPCs and a decrease in surface AMPA expression [24, 29–31]. In epilepsy-related studies, neuronal hyperexcitability and decreased inhibition have been frequently observed in iPSC-derived neurons from children with mutations associated with drug-resistant epilepsy [32–35] and multifocal epilepsy [20]. Our study presents for the first time a human model of the A350V *IQSEC2* mutation and provides a thorough characterization of the different stages of the development of these mutant neurons, providing mechanistic explanations for the behavioral phenotype of this disease in humans.

## Supplementary information

Supplementary

Supplementary Table S1.

Supplementary Table S2.

Supplementary Table S5.

Supplementary Table S6.

Supplementary Table S7.

Supplementary Figure S8.

Supplementary Table S9.

Supplementary Table S10.

Supplementary Table S11.

Supplementary Table S12.

Supplementary Figure S13.

Supplementary Figure S14.

## Data Availability

The data is available at https://usegalaxy.org/u/borisbrant/h/iqsec
