# Supplementary material for: IQSEC2 mutation associated with epilepsy, intellectual disability, and autism results in hyperexcitability of patient-derived neurons and deficient synaptic transmission
Source: Mol Psychiatry. 2021 Sep 17;26(12):7498–508. doi: 10.1038/s41380-021-01281-0 (PMC8873005; doi:10.1038/s41380-021-01281-0)
Supplement: Supplementary file 1 — Supplementary [file 41380_2021_1281_MOESM1_ESM.docx]

**Supplementary Material**

**Supplementary Table S1**. Differentially expressed genes between patient derived *IQSEC2-*mutated DG granule neurons and CRISPR-Cas9 corrected isogeneic control DG granule neurons at 5 weeks post differentiation.

**Supplementary Table S2.** Differentially expressed genes between patient derived *IQSEC2* mutated DG granule neurons and CRISPR corrected isogeneic control DG granule neurons at 7 weeks post differentiation.

**Supplementary Table 3.** Down regulated GO terms in the RNA sequencing analysis of *IQSEC2* mutated DG granule neurons compared to controls at 11 weeks post differentiation.

**Supplementary Table 4.** Down regulated functional categories in the RNA sequencing analysis of *A350V* *IQSEC2* mutated DG granule neurons compared to controls at 11 weeks post differentiation.

**Supplementary Table S5.** Differentially expressed genes between *IQSEC2*-mutated DG granule neurons compared to controls at 11 weeks post differentiation.

Supplementary Table S6. A list of the genes in the dysregulated pathway “transmission of nerve impulse” between *IQSEC2*-mutated DG granule neurons and controls at 11 weeks post differentiation.

Supplementary Table S7. A list of the genes in the dysregulated pathway “synaptic transmission” between IQSEC2-mutated DG granule neurons and controls at 11 weeks post differentiation.

Supplementary Figure S8. Characterization of iPSC lines. a-b. Demonstration of a normal karyotype for the two patient derived iPSC clones (C1287 clones 1 and 3) used throughout the study. c. Both patient derived iPSC clones express pluripotency markers. d. Sequence chromatograms of the hIQSEC2 locus in patient derived iPSC line C1471 C3 (upper graph) and CRISPR corrected line C1287 C6 (lower graph). e. The results were confirmed via next generation sequencing (NGS) (upper graph) patient iPSC line C1471 C3 and CRISPR corrected line C1287 C6 (lower graph).

**Supplementary Table S9.** Upregulated GO terms in the RNA sequencing analysis of A350V *IQSEC2* mouse hippocampi compared to wild type mouse hippocampi.

**Supplementary Table S10.** Upregulated functional categories and KEGG pathways in the RNA sequencing analysis of A350V *IQSEC2* mouse hippocampi compared to wild type mouse hippocampi.

**Supplementary Table S11.** Down regulated GO terms in the RNA sequencing analysis of A350V *IQSEC2* mouse hippocampi compared to wild type mouse hippocampi.

**Supplementary Table S12.** Downregulated functional categories and KEGG pathways in the RNA sequencing analysis A350V *IQSEC2* mouse hippocampi compared to wild type mouse hippocampi.

**Supplementary Figure S13.** Network activity is reduced in A350V *IQEC2*-mutated human neuronal cultures compared to CRISPR-corrected controls at 11 weeks post-differentiation. a. Burst duration is significantly shorter in *IQSEC2*-mutated neuronal cultures compared to corrected control neuronal cultures. b. The rate of bursts is significantly lower in *IQSEC2*-mutated neuronal cultures compared to corrected control neuronal cultures. c. The interval between bursts is significantly longer in *IQSEC2*-mutated neuronal cultures compared to corrected control neuronal cultures. d. The resting membrane potential is not significantly different between A350V *IQSEC2*-mutated neurons and CRISPR-corrected control neurons (p=0.31).

**Supplementary Figure S14.** Transcriptional similarities between 11 weeks post-differentiation human A350V *IQSEC2* hippocampal neurons and mice A350V *IQSEC2* hippocampal tissue. a. Five top down regulated Gene ontology Functional categories in 11weeks post-differentiation human DG granule neurons compared to CRISPR-corrected controls. b. Five top down regulated Gene ontology Functional categories A350V *IQSEC2* mice hippocampal tissue compared to WT mice. c. Five top up regulated Gene ontology Functional categories in 11 weeks post-differentiation human DG granule neurons compared to CRISPR-corrected controls. b. Five top up regulated Gene ontology Functional categories A350V *IQSEC2* mice hippocampal tissue compared to WT mice.

**Supplementary Table 3**

| **GO Down in IQsec2** | #genes | Log10(p) | Fold | FDR |
| --- | --- | --- | --- | --- |
| GO:0030198~extracellular matrix organization | 38 | -16.12 | 4.99 | 1.9984E-13 |
| GO:0043062~extracellular structure organization | 45 | -13.91 | 3.77 | 2.28484E-11 |
| GO:0007155~cell adhesion | 109 | -13.40 | 2.13 | 7.36522E-11 |
| GO:0022610~biological adhesion | 109 | -13.36 | 2.12 | 8.12017E-11 |
| GO:0042127~regulation of cell proliferation | 107 | -9.46 | 1.86 | 6.42674E-07 |
| GO:0016477~cell migration | 52 | -9.24 | 2.57 | 1.06506E-06 |
| GO:0006928~cell motion | 73 | -8.69 | 2.10 | 3.74401E-06 |
| GO:0051674~localization of cell | 53 | -8.04 | 2.36 | 1.67184E-05 |
| GO:0048870~cell motility | 53 | -8.04 | 2.36 | 1.67184E-05 |
| GO:0001501~skeletal system development | 53 | -7.46 | 2.27 | 6.30572E-05 |
| GO:0051270~regulation of cell motion | 38 | -7.30 | 2.69 | 9.23216E-05 |
| GO:0031589~cell-substrate adhesion | 25 | -6.99 | 3.48 | 0.000189688 |
| GO:0007167~enzyme linked receptor protein signaling pathway | 54 | -6.86 | 2.16 | 0.000255689 |
| GO:0032989~cellular component morphogenesis | 59 | -6.55 | 2.03 | 0.00052295 |
| GO:0030199~collagen fibril organization | 13 | -6.45 | 6.12 | 0.000655368 |
| GO:0000902~cell morphogenesis | 54 | -6.29 | 2.07 | 0.000941186 |
| GO:0030334~regulation of cell migration | 33 | -6.27 | 2.67 | 0.000981404 |
| GO:0045597~positive regulation of cell differentiation | 40 | -6.24 | 2.38 | 0.001046092 |
| GO:0007507~heart development | 38 | -6.07 | 2.41 | 0.001562741 |
| GO:0051094~positive regulation of developmental process | 45 | -6.05 | 2.21 | 0.001643656 |
| GO:0010033~response to organic substance | 89 | -5.97 | 1.69 | 0.001972522 |
| GO:0040012~regulation of locomotion | 35 | -5.92 | 2.49 | 0.002210017 |
| GO:0007160~cell-matrix adhesion | 22 | -5.91 | 3.37 | 0.002281926 |
| GO:0030182~neuron differentiation | 61 | -5.79 | 1.90 | 0.002968823 |
| GO:0007610~behavior | 64 | -5.76 | 1.86 | 0.00317964 |
| GO:0048729~tissue morphogenesis | 33 | -5.65 | 2.50 | 0.004144957 |
| GO:0001944~vasculature development | 41 | -5.63 | 2.23 | 0.004307363 |
| GO:0000904~cell morphogenesis involved in differentiation | 40 | -5.54 | 2.24 | 0.005336178 |
| GO:0001568~blood vessel development | 40 | -5.49 | 2.23 | 0.005910185 |
| GO:0009725~response to hormone stimulus | 52 | -5.19 | 1.93 | 0.01194492 |
| GO:0009611~response to wounding | 68 | -5.17 | 1.75 | 0.012446827 |
| GO:0008285~negative regulation of cell proliferation | 51 | -5.06 | 1.93 | 0.016117443 |
| GO:0048545~response to steroid hormone stimulus | 33 | -5.04 | 2.35 | 0.016940525 |
| GO:0048754~branching morphogenesis of a tube | 17 | -4.89 | 3.57 | 0.023451289 |
| GO:0035239~tube morphogenesis | 25 | -4.85 | 2.69 | 0.026132005 |
| GO:0008284~positive regulation of cell proliferation | 55 | -4.65 | 1.81 | 0.04118636 |
| GO:0009719~response to endogenous stimulus | 54 | -4.62 | 1.82 | 0.044413181 |
| GO:0035295~tube development | 35 | -4.59 | 2.17 | 0.046990784 |
| GO:0001503~ossification | 23 | -4.58 | 2.73 | 0.048263676 |

**Supplementary Table 4**

| **Functional categories Down in IQsec2** | #genes | Log10(p) | Fold | FDR |
| --- | --- | --- | --- | --- |
| signal | 377 | -30.78 | 1.75 | 2.48E-28 |
| glycoprotein | 462 | -30.60 | 1.62 | 3.73E-28 |
| extracellular matrix | 66 | -22.55 | 4.13 | 4.21E-20 |
| disease mutation | 180 | -12.31 | 1.71 | 7.34E-10 |
| hydroxylation | 26 | -11.87 | 5.53 | 2.02E-09 |
| cell adhesion | 69 | -11.18 | 2.47 | 9.84E-09 |
| membrane | 525 | -10.87 | 1.27 | 2.02E-08 |
| Secreted | 183 | -10.87 | 1.64 | 2.02E-08 |
| disulfide bond | 278 | -10.14 | 1.44 | 1.08E-07 |
| trimer | 15 | -9.88 | 8.71 | 1.95E-07 |
| hydroxylysine | 15 | -8.58 | 7.31 | 3.91E-06 |
| triple helix | 15 | -8.58 | 7.31 | 3.91E-06 |
| egf-like domain | 42 | -8.27 | 2.76 | 7.99E-06 |
| collagen | 25 | -8.08 | 3.97 | 1.23E-05 |
| hydroxyproline | 15 | -7.38 | 6.12 | 6.17E-05 |
| golgi apparatus | 74 | -6.85 | 1.90 | 0.000212 |
| phosphoprotein | 566 | -6.48 | 1.18 | 0.00049 |
| polymorphism | 848 | -6.34 | 1.11 | 0.000682 |
| transmembrane protein | 77 | -6.27 | 1.81 | 0.000792 |
| endoplasmic reticulum | 83 | -6.20 | 1.76 | 0.000936 |
| Growth factor binding | 9 | -5.54 | 8.49 | 0.00426 |
| calcium | 86 | -4.96 | 1.62 | 0.016258 |
| lysosome | 26 | -4.82 | 2.63 | 0.022307 |
| transmembrane | 394 | -4.76 | 1.20 | 0.025931 |
| pyroglutamic acid | 13 | -4.50 | 4.27 | 0.047086 |

**Supplementary Methods**

**Electrophysiology**

Neurons on glass or plastic coverslips were transferred to a recording chamber in standard recording medium containing (in mM) 10 HEPES, 4 KCl, 2 CaCl2,1 MgCl2, 139 NaCl, and 10 D-glucose (310 mOsm, pH 7.4). Patch electrodes were filled with internal solutions containing (in mM) 130 K-gluconate, 6 KCl, 4 NaCl, 10 Na-HEPES, 0.2 K-EGTA, 0.3 GTP, 2 Mg-ATP, 0.2 cAMP, 10 Dglucose, 0.15% biocytin and 0.06% rhodamine. The pH and osmolarity of the internal solution were brought close to physiological conditions (pH 7.3, 290–300 mOsmol). Signals were amplified with a Multiclamp700B amplifier and recorded with Clampex 10.2 software (Axon Instruments). Data were acquired at a sampling rate of 20 kHz and analyzed using Clampfit-10 and the software package Matlab (2018b, The MathWorks Inc., Natick, MA, 2000). All measurements were conducted at room temperature.

**Analysis of electrophysiological recordings**

***Total evoked action potentials***

To define excitability, we used a quantification measure that was similar to our previous studies ^13-15^. Cells were typically held in current clamp mode near -60 mV with a steady holding current, and current injections were given starting 5 pA below the steady holding current, in 3 pA steps of 400 ms in duration. A total of 35 depolarization steps were given. Neurons that needed a current injection of more than 50 pA to be held at -60 mV were discarded from the analysis. The total number of action potentials was counted in 35 depolarization steps, starting from the first depolarization step, which was 10 pA below the current and caused the membrane potential to be at -60 mV (typically around 0).

***Maximum evoked action potentials***

The maximum evoked potentials were measured similar to that of the total evoked potentials, but rather than taking the sum of the evoked potentials, this measure was used to indicate the maximum total evoked potentials in any of the 400 ms current steps (the current injection step that gives the maximum evoked potentials over 35 current injection steps).

***Spike shape analysis***

When injecting with minimal current needed for evoking an action potential, we analyzed the first evoked action potential. Spike threshold was the membrane potential at which the slope of the depolarizing membrane potential increased drastically, resulting in an action potential (the first maximum in the second derivative of the voltage vs. time). The fast after hyperpolarization (AHP) amplitude was calculated as the difference between the threshold for spiking and the value of the membrane potential 5 ms after the potential returned to cross the threshold value at the end of the action potential. The spike amplitude (or spike height) was calculated as the difference between the maximum membrane potential during a spike and the threshold. Action potential width was calculated as the time it took the membrane potential to reach half the spike amplitude in the rising part of the spike to the descending part of the spike (Full Width at Half Maximum, FWHM).

***Cell capacitance***

The cell capacitance was measured by the Clampex SW at a 100 Hz update rate.

***Resting membrane Potential***

The resting membrane potentials were calculated in current clamp mode at 0 pA current. Cells with holding currents larger than 20 pA were discarded from this analysis.

***Sodium and potassium currents***

The sodium and potassium currents were acquired in voltage clamp mode. Cells were held at −60 mV, and voltage steps of 400 ms were made in the range of −90 mV to 80 mV. Currents were normalized by the cell capacitance (changing the units from pA to pA/pF), by an automatic Matlab script. The amplitude of the sodium currents was calculated as the amplitude of the inward currents in voltage clamp mode in different test potentials. We measured the fast potassium current by the maximum current immediately following a depolarization step, typically within a time window of a few milliseconds. The slow potassium currents were obtained at the end of the 400-ms depolarization step.

***Synaptic activity***

The synaptic activity was measured in a voltage clamp of -60 mV with 40 μM bicuculline in the recording medium. Synaptic currents were recorded and analyzed by a Matlab script.

***Network activity***

We analyzed thy synaptic activity (at 11 weeks of differentiation) and defined the end of a network burst where there was a quiescence of activity for at least 250 ms. The burst duration, bursting rate, and interburst itervals were quantified for each of the recorded neurons.

**RNA sequencing and analysis for iPSC-derived FACS-sorted DG granule neurons**

Sequenced reads were quality-tested using FASTQC ^14^ v0.11.5 and aligned to the hg38 (Lander et al., 2001) human genome using Galaxy. Mapping was carried out using default parameters, filtering non-canonical introns and allowing up to 10 mismatches per read and only keeping uniquely mapped reads. The genome index was constructed using the gene annotation supplied with the hg38 Illumina iGenomes collection (Illumina, 2015). Raw or FPKM (Fragments Per Kilobase Million) gene expression was quantified across all gene exons with SeqMonk using the top-expressed isoform as proxy for gene expression, and differential gene expression was carried out on the raw counts using R-based *DESeq2* algorithm. For each individual disease type, differentially expressed genes were defined as having a false discovery rate (FDR) <0.05 when comparing 2 experimental conditions. Gene Ontology (GO) enrichment test was performed using the program DAVID Bioinformatics Resources 6.8 ^15^. Overrepresentation of GO terms was determined by FDR < 0.05 using the corrected Benjamini & Yakutieli method for multiple testing correction ^16^.

**RNA sequencing of hippocampi of A350V IQSEC2 vs wild type IQSEC2 mice**

The creation of A350V *IQSEC2* mice in a C57/Bl6J background was previously described as well as the PCR based genotyping strategy used to distinguish the mutant allele from the wild type allele^10^. Mice were housed and maintained at the Technion Faculty of Medicine germ free animal facility under the approval of the Technion institutional animal care and use committees (IL1691117).

RNA sequencing was performed on post-natal day 16 for RNA extracted from hippocampi of A350V *IQSEC2* and wild type mice at the Genomics Center of the Biomedical Core Facility (BCF) of the Technion Faculty of Medicine. Further information is provided in supplementary methods. This specific time point was chosen as it is one day prior to when the *IQSEC2* mice begin to have seizures. The analysis was performed on hippocampal RNA obtained from 4 males with the A350V *IQSEC2* mutation and 3 wild type males from the same litter from a mating between a female heterozygous for the A350V *IQSEC2* mutation and a wild type male. Total RNA was extracted from these 7 mouse hippocampal tissue samples using the Qiacube (Qiagen) with the RNeasy kit (cat no. 74106). Seven RNA sequencing libraries were constructed simultaneously according to the manufacturers protocol (NEBNExt Ultra II Directional RNA Library Prep Kit for Ilumina, cat no E7760) using 800 ng of total RNA as starting material. RNA sequencing data was generated on an Illumina NextSeq500, 75 cycles (single read), high output mode (Ilumina cat 20024906). Statistical analysis was performed using DEseq2 package (version 1.20.0). Functional annotation was performed with the Database for Annotation, Visualization and Integrated Discovery (DAVID).
