# Supplementary figures and images for: IQSEC2 mutation associated with epilepsy, intellectual disability, and autism results in hyperexcitability of patient-derived neurons and deficient synaptic transmission
Source: Mol Psychiatry. 2021 Sep 17;26(12):7498–508. doi: 10.1038/s41380-021-01281-0 (PMC8873005; doi:10.1038/s41380-021-01281-0)

## Slide 1
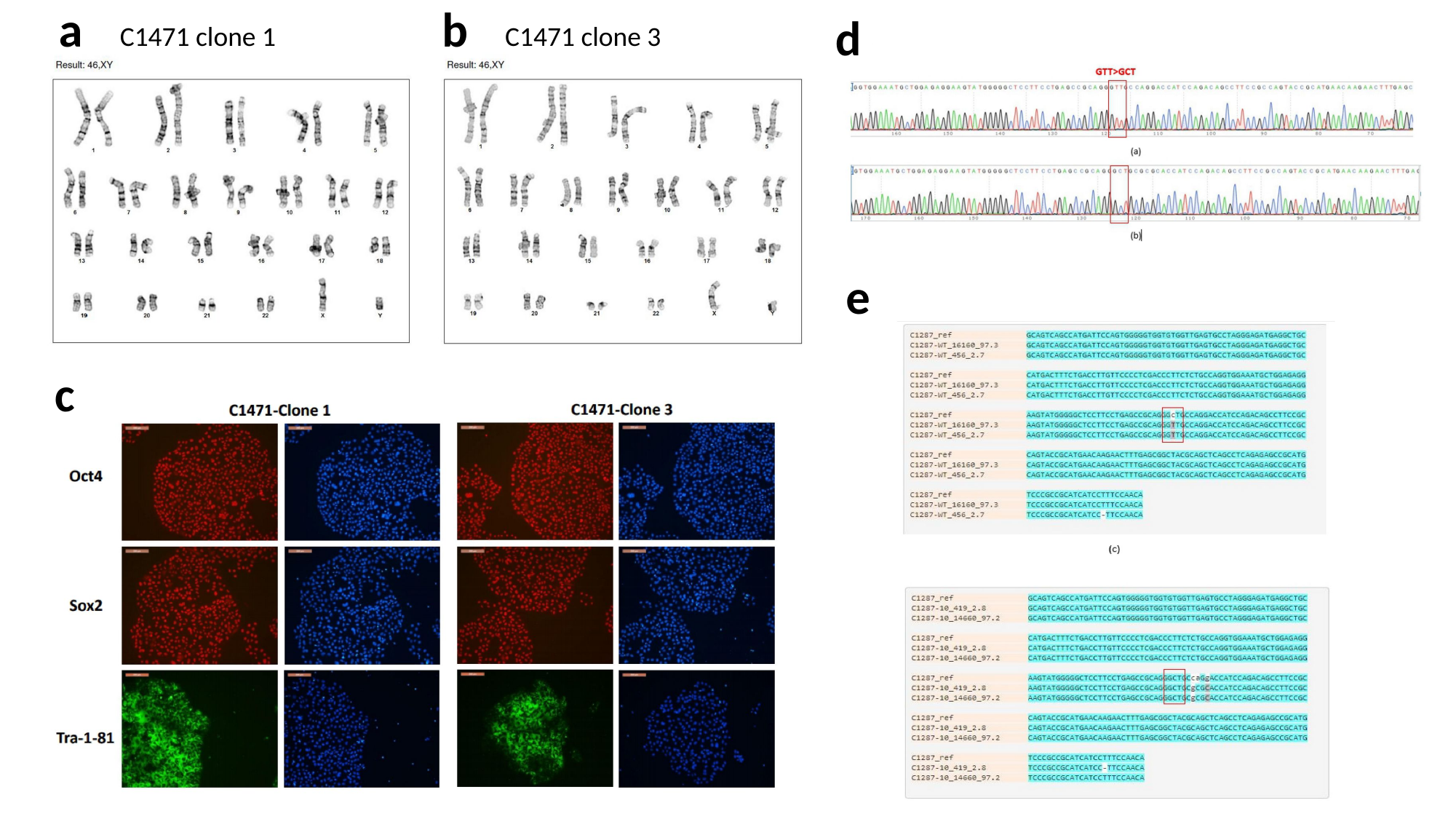

a
b
d
C1471 clone 1
C1471 clone 3
e
c

Supplement: Supplementary file 7 — Supplementary Figure S8. [file 41380_2021_1281_MOESM7_ESM.pptx]

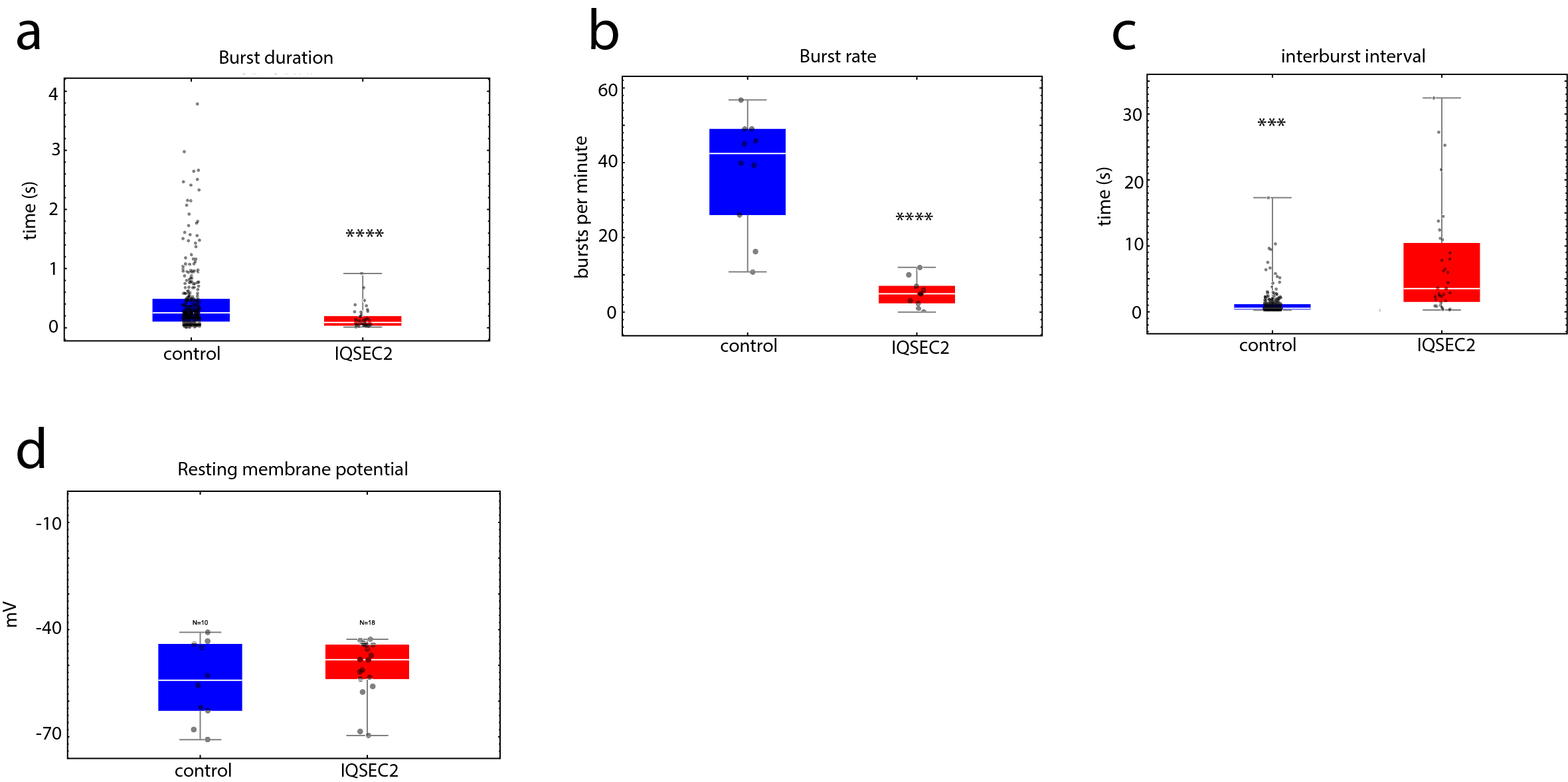

Supplement: Supplementary file 12 — Supplementary Figure S13. [file 41380_2021_1281_MOESM12_ESM.jpg]

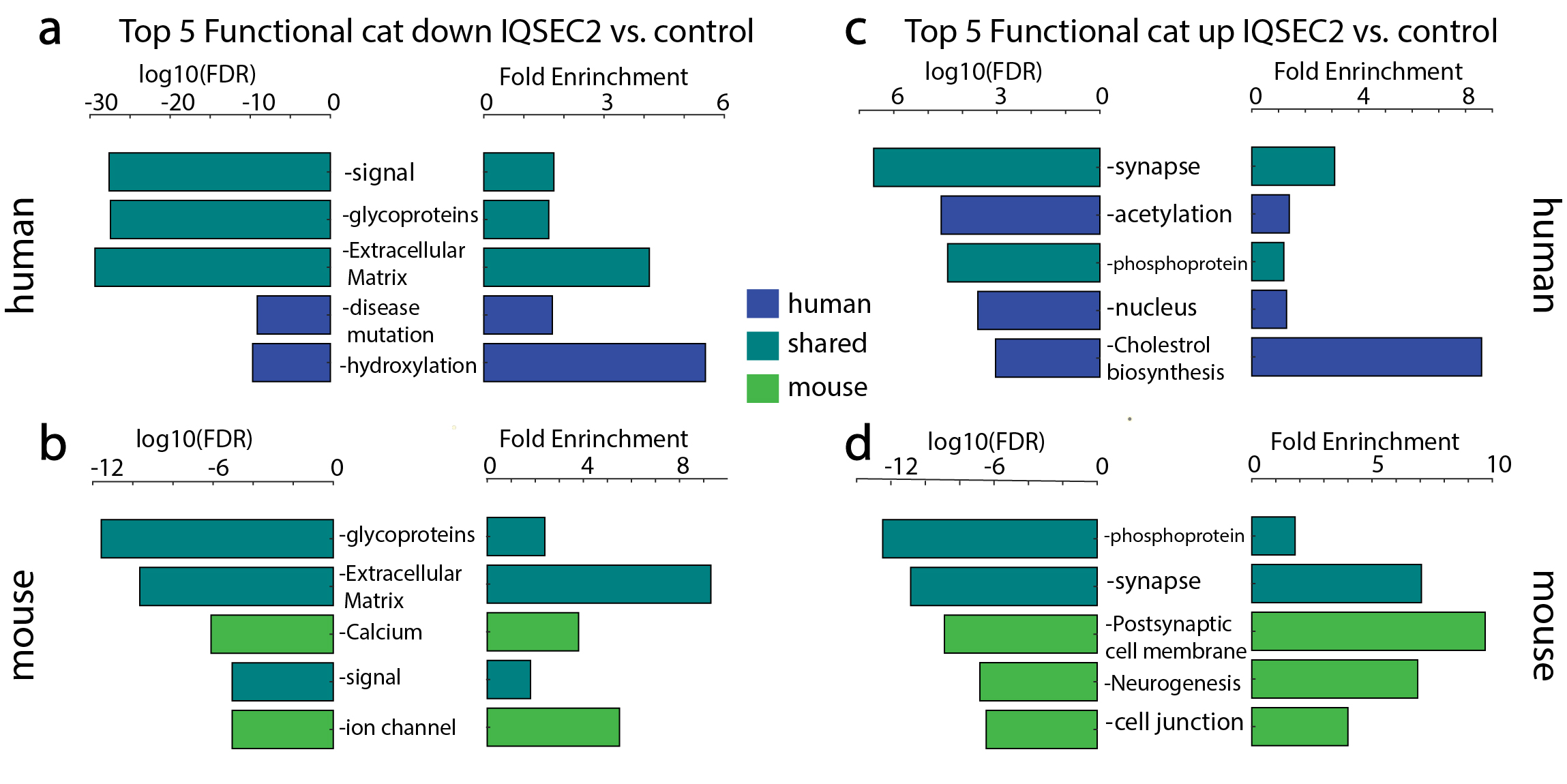

Supplement: Supplementary file 13 — Supplementary Figure S14. [file 41380_2021_1281_MOESM13_ESM.jpg]
