# Supplementary material for: IQSEC2 mutation associated with epilepsy, intellectual disability, and autism results in hyperexcitability of patient-derived neurons and deficient synaptic transmission
Source: Mol Psychiatry. 2021 Sep 17;26(12):7498–508. doi: 10.1038/s41380-021-01281-0 (PMC8873005; doi:10.1038/s41380-021-01281-0)
Supplement: Supplementary file 8 — Supplementary Table S9. [file 41380_2021_1281_MOESM8_ESM.docx]

| **Go Terms Up in Mouse** | #genes | Log10 (pValue) | Fold | FDR |
| --- | --- | --- | --- | --- |
| GO:0007399~nervous system development | 22 | -8.60 | 5.072995307 | 3.07794E-06 |
| GO:0035556~intracellular signal transduction | 22 | -8.15 | 4.781298077 | 4.34576E-06 |
| GO:0006816~calcium ion transport | 12 | -6.17 | 7.398527005 | 0.000281233 |
| GO:0016310~phosphorylation | 22 | -5.11 | 3.125031423 | 0.002394474 |
| GO:0007268~chemical synaptic transmission | 11 | -4.53 | 5.559648927 | 7.38E-03 |
| GO:0006468~protein phosphorylation | 20 | -4.44 | 3.018496261 | 7.56E-03 |
| GO:0048666~neuron development | 7 | -4.29 | 10.49187666 | 9.14E-03 |
| GO:0061003~positive regulation of dendritic spine morphogenesis | 5 | -4.15 | 21.73317308 | 1.08E-02 |
| GO:0048167~regulation of synaptic plasticity | 6 | -3.95 | 12.41895604 | 1.55E-02 |
| GO:0048013~ephrin receptor signaling pathway | 6 | -3.90 | 12.13014311 | 1.57E-02 |
| GO:0043087~regulation of GTPase activity | 7 | -3.79 | 8.570828819 | 1.82E-02 |
| GO:0007264~small GTPase mediated signal transduction | 11 | -3.39 | 4.051947523 | 4.17E-02 |
| GO:0070588~calcium ion transmembrane transport | 7 | -3.28 | 6.915100524 | 4.96E-02 |
