# Supplementary material for: IQSEC2 mutation associated with epilepsy, intellectual disability, and autism results in hyperexcitability of patient-derived neurons and deficient synaptic transmission
Source: Mol Psychiatry. 2021 Sep 17;26(12):7498–508. doi: 10.1038/s41380-021-01281-0 (PMC8873005; doi:10.1038/s41380-021-01281-0)
Supplement: Supplementary file 9 — Supplementary Table S10. [file 41380_2021_1281_MOESM9_ESM.docx]

| **Functional Categories Up in Mouse** | #genes | Log10 (pValue) | Fold | FDR |
| --- | --- | --- | --- | --- |
| Phosphoprotein | 134 | -14.71 | 1.773296573 | 3.28237E-13 |
| Synapse | 25 | -12.79 | 7.058823529 | 1.37912E-11 |
| Postsynaptic cell membrane | 17 | -10.62 | 9.736363636 | 1.33924E-09 |
| Neurogenesis | 17 | -8.45 | 6.937651822 | 1.51013E-07 |
| Cell junction | 26 | -7.98 | 3.964901664 | 3.52E-07 |
| Cell projection | 26 | -7.77 | 3.865486726 | 4.82E-07 |
| Calmodulin-binding | 12 | -6.85 | 8.702158273 | 3.40E-06 |
| Cytoplasm | 76 | -6.37 | 1.739509537 | 8.96E-06 |
| Calcium | 26 | -6.14 | 3.16904474 | 1.37E-05 |
| Guanine-nucleotide releasing factor | 11 | -6.07 | 8.336842105 | 1.43E-05 |
| Cell membrane | 67 | -6.00 | 1.796648045 | 1.54E-05 |
| Kinase | 23 | -5.65 | 3.279207921 | 3.14E-05 |
| Alternative splicing | 78 | -5.59 | 1.64519774 | 3.35E-05 |
| Glycoprotein | 65 | -5.13 | 1.717431193 | 8.96E-05 |
| Ion channel | 14 | -4.49 | 4.2 | 3.63E-04 |
| Calcium transport | 8 | -4.41 | 8.670967742 | 4.07E-04 |
| Actin-binding | 12 | -4.35 | 4.8 | 4.23E-04 |
| Membrane | 116 | -4.35 | 1.346631349 | 4.23E-04 |
| ATP-binding | 30 | -4.09 | 2.218635363 | 7.19E-04 |
| Ligand-gated ion channel | 7 | -4.07 | 9.665753425 | 7.19E-04 |
| Serine/threonine-protein kinase | 14 | -3.68 | 3.484444444 | 1.68E-03 |
| GTPase activation | 9 | -3.65 | 5.565644172 | 1.72E-03 |
| Nucleotide-binding | 34 | -3.58 | 1.953933865 | 1.91E-03 |
| Developmental protein | 23 | -3.55 | 2.375409836 | 2.00E-03 |
| Calcium channel | 6 | -3.43 | 9.75483871 | 2.49E-03 |
| Sarcoplasmic reticulum | 5 | -3.28 | 13.26315789 | 3.42E-03 |
| Ion transport | 16 | -2.88 | 2.60549273 | 8.26E-03 |
| Coiled coil | 48 | -2.86 | 1.572951886 | 8.37E-03 |
| Zinc | 36 | -2.83 | 1.728823249 | 8.66E-03 |
| Cytoskeleton | 22 | -2.64 | 2.066728798 | 1.29E-02 |
| Transferase | 29 | -2.43 | 1.767351874 | 2.01E-02 |
| SH3 domain | 8 | -2.32 | 3.876923077 | 2.50E-02 |
| Zinc-finger | 27 | -2.19 | 1.739041534 | 3.32E-02 |
| Metal-binding | 48 | -2.03 | 1.425154639 | 4.65E-02 |

| Pathways Up in Mouse | #genes | Log10 (pValue) | Fold | FDR |
| --- | --- | --- | --- | --- |
| mmu04020: Calcium signaling pathway | 17 | -9.48 | 7.727364066 | 4.01471E-08 |
| mmu04921: Oxytocin signaling pathway | 15 | -8.61 | 8.181914894 | 1.50821E-07 |
| mmu04720: Long-term potentiation | 10 | -7.11 | 12.39684075 | 3.1896E-06 |
| mmu04713: Circadian entrainment | 11 | -6.62 | 9.183782023 | 7.28844E-06 |
| mmu05031: Amphetamine addiction | 9 | -5.91 | 10.99063195 | 2.99E-05 |
| mmu04725: Cholinergic synapse | 9 | -4.21 | 6.516569384 | 1.13E-03 |
| mmu04724: Glutamatergic synapse | 9 | -4.16 | 6.403237743 | 1.13E-03 |
| mmu04925: Aldosterone synthesis and secretion | 8 | -4.13 | 7.611083622 | 1.13E-03 |
| mmu04024: cAMP signaling pathway | 11 | -3.91 | 4.56858192 | 1.67E-03 |
| mmu04360: Axon guidance | 9 | -3.81 | 5.708312716 | 1.90E-03 |
| mmu05231: Choline metabolism in cancer | 8 | -3.69 | 6.480724668 | 2.19E-03 |
| mmu04971: Gastric acid secretion | 7 | -3.66 | 7.95463948 | 2.19E-03 |
| mmu04810: Regulation of actin cytoskeleton | 11 | -3.63 | 4.225402058 | 2.19E-03 |
| mmu04911: Insulin secretion | 7 | -3.24 | 6.659698169 | 5.01E-03 |
| mmu04730: Long-term depression | 6 | -3.09 | 8.047785141 | 6.52E-03 |
| mmu04010: MAPK signaling pathway | 11 | -3.07 | 3.585699754 | 6.52E-03 |
| mmu04070: Phosphatidylinositol signaling system | 7 | -2.96 | 5.904474665 | 7.70E-03 |
| mmu04728: Dopaminergic synapse | 8 | -2.94 | 4.88472531 | 7.70E-03 |
| mmu05033: Nicotine addiction | 5 | -2.88 | 10.22739362 | 8.43E-03 |
| mmu04723: Retrograde endocannabinoid signaling | 7 | -2.83 | 5.560524685 | 8.83E-03 |
| mmu04310: Wnt signaling pathway | 8 | -2.82 | 4.642221216 | 8.83E-03 |
| mmu04261: Adrenergic signaling in cardiomyocytes | 8 | -2.80 | 4.609529518 | 8.83E-03 |
| mmu05014: Amyotrophic lateral sclerosis (ALS) | 5 | -2.49 | 8.02148519 | 1.72E-02 |
| mmu04666: Fc gamma R-mediated phagocytosis | 6 | -2.47 | 5.844224924 | 1.72E-02 |
| mmu04912: GnRH signaling pathway | 6 | -2.38 | 5.578578337 | 1.98E-02 |
| mmu04015: Rap1 signaling pathway | 9 | -2.37 | 3.440992245 | 1.98E-02 |
| mmu00561: Glycerolipid metabolism | 5 | -2.29 | 7.053374908 | 2.33E-02 |
| mmu04068: FoxO signaling pathway | 7 | -2.26 | 4.274134646 | 2.41E-02 |
| mmu04916: Melanogenesis | 6 | -2.17 | 4.958736299 | 2.86E-02 |
| mmu04972: Pancreatic secretion | 6 | -2.15 | 4.909148936 | 2.88E-02 |
| mmu04080: Neuroactive ligand-receptor interaction | 10 | -2.14 | 2.870847331 | 2.88E-02 |
| mmu04520: Adherens junction | 5 | -1.96 | 5.681885343 | 4.17E-02 |
| mmu04114: Oocyte meiosis | 6 | -1.95 | 4.383168693 | 4.17E-02 |
| mmu04022: cGMP-PKG signaling pathway | 7 | -1.86 | 3.513705783 | 4.83E-02 |
| mmu04970: Salivary secretion | 5 | -1.858415059 | 5.312931749 | 4.83E-02 |
