# Supplementary material for: IQSEC2 mutation associated with epilepsy, intellectual disability, and autism results in hyperexcitability of patient-derived neurons and deficient synaptic transmission
Source: Mol Psychiatry. 2021 Sep 17;26(12):7498–508. doi: 10.1038/s41380-021-01281-0 (PMC8873005; doi:10.1038/s41380-021-01281-0)
Supplement: Supplementary file 10 — Supplementary Table S11. [file 41380_2021_1281_MOESM10_ESM.docx]

| **GO Terms Down in Mouse** | #genes | Log10 (pValue) | Fold | FDR |
| --- | --- | --- | --- | --- |
| GO:0006811~ion transport | 18 | -4.33E+00 | 3.202999528 | 0.049349401 |
