# Supplementary material for: IQSEC2 mutation associated with epilepsy, intellectual disability, and autism results in hyperexcitability of patient-derived neurons and deficient synaptic transmission
Source: Mol Psychiatry. 2021 Sep 17;26(12):7498–508. doi: 10.1038/s41380-021-01281-0 (PMC8873005; doi:10.1038/s41380-021-01281-0)
Supplement: Supplementary file 11 — Supplementary Table S12. [file 41380_2021_1281_MOESM11_ESM.docx]

| **Functional categories Down in Mouse** | #genes | Log10 (pValue) | Fold | FDR |
| --- | --- | --- | --- | --- |
| Glycoprotein | 79 | -1.38E+01 | 2.384017138 | 2.65E-12 |
| Extracellular matrix | 19 | -1.16E+01 | 9.308132628 | 2.17E-10 |
| Calcium | 27 | -7.87E+00 | 3.758677625 | 7.91E-07 |
| Signal | 71 | -6.67E+00 | 1.799253831 | 8.98E-06 |
| Ion channel | 16 | -6.60E+00 | 5.482233503 | 8.98E-06 |
| Cell junction | 22 | -6.51E+00 | 3.831757758 | 9.20E-06 |
| Disulfide bond | 54 | -6.17E+00 | 1.990029703 | 1.73E-05 |
| Ion transport | 20 | -5.71E+00 | 3.719770713 | 4.32E-05 |
| Secreted | 35 | -5.53E+00 | 2.391360014 | 5.25E-05 |
| Synapse | 15 | -5.53E+00 | 4.837264855 | 5.25E-05 |
| Collagen | 8 | -5.04E+00 | 10.83547328 | 1.45E-04 |
| Voltage-gated channel | 9 | -4.61E+00 | 7.618692147 | 3.64E-04 |
| Potassium transport | 8 | -4.39E+00 | 8.607618957 | 5.54E-04 |
| Cell adhesion | 15 | -4.31E+00 | 3.76231711 | 6.21E-04 |
| Potassium | 8 | -4.07E+00 | 7.675126904 | 9.96E-04 |
| Phosphoprotein | 92 | -3.91E+00 | 1.390531066 | 1.36E-03 |
| Pyrrolidone carboxylic acid | 5 | -2.77E+00 | 9.75651725 | 1.77E-02 |
| Transmembrane helix | 80 | -2.63E+00 | 1.327493843 | 2.31E-02 |
| Transmembrane | 80 | -2.60E+00 | 1.32424907 | 2.36E-02 |
| Potassium channel | 5 | -2.57E+00 | 8.591559967 | 2.40E-02 |
| Immunoglobulin domain | 12 | -2.49E+00 | 2.872188862 | 2.66E-02 |
| Lipoprotein | 16 | -2.48E+00 | 2.361577509 | 2.66E-02 |
| Membrane | 95 | -2.46E+00 | 1.259594131 | 2.69E-02 |
| Ligand-gated ion channel | 5 | -2.43E+00 | 7.885404353 | 2.69E-02 |
| Transport | 29 | -2.42E+00 | 1.756275751 | 2.69E-02 |
| Postsynaptic cell membrane | 7 | -2.36E+00 | 4.578910937 | 2.96E-02 |
| Alternative splicing | 58 | -2.32E+00 | 1.397229631 | 3.17E-02 |
| Hydroxylation | 5 | -2.14E+00 | 6.541301338 | 4.53E-02 |
| Cell membrane | 47 | -2.11E+00 | 1.439469132 | 4.77E-02 |

| Pathways Down in Mouse | #genes | Log10 (pValue) | Fold | FDR |
| --- | --- | --- | --- | --- |
| mmu04974: Protein digestion and absorption | 7 | -3.83E+00 | 8.497001263 | 0.017151024 |
| mmu05033: Nicotine addiction | 5 | -3.32E+00 | 13.35243056 | 0.027268199 |
| mmu04512: ECM-receptor interaction | 6 | -2.90E+00 | 7.283143939 | 0.03817542 |
| mmu00534: Glycosaminoglycan biosynthesis - heparan sulfate / heparin | 4 | -2.88E+00 | 17.80324074 | 0.03817542 |
